# Global Impact of Mass Vaccination Campaigns on Circulating Type 2 Vaccine-Derived Poliovirus Outbreaks: An Interrupted Time-Series Analysis

**DOI:** 10.1093/infdis/jiae614

**Published:** 2025-01-28

**Authors:** Laura V Cooper, Ananda S Bandyopadhyay, Nicholas C Grassly, Elizabeth J Gray, Arie Voorman, Simona Zipursky, Isobel M Blake

**Affiliations:** School of Public Health, Medical Research Council Centre for Global Infectious Disease Analysis, Imperial College London, London, United Kingdom; Bill and Melinda Gates Foundation, Polio Team, Seattle, Washington, USA; School of Public Health, Medical Research Council Centre for Global Infectious Disease Analysis, Imperial College London, London, United Kingdom; School of Public Health, Medical Research Council Centre for Global Infectious Disease Analysis, Imperial College London, London, United Kingdom; Bill and Melinda Gates Foundation, Polio Team, Seattle, Washington, USA; Bill and Melinda Gates Foundation, Polio Team, Seattle, Washington, USA; School of Public Health, Medical Research Council Centre for Global Infectious Disease Analysis, Imperial College London, London, United Kingdom

**Keywords:** poliovirus, vaccine, surveillance, mass vaccination campaigns, time-series analysis

## Abstract

**Background:**

Between 2016 and 2023, 3248 cases of circulating vaccine-derived type 2 poliomyelitis (cVDPV2) were reported globally and supplementary immunization activities (SIAs) with monovalent type 2 oral poliovirus vaccine (mOPV2) and novel type 2 oral poliovirus vaccine (nOPV2) targeted an estimated 356 and 525 million children, respectively. This analysis estimates the community-level impact of nOPV2 relative to mOPV2 SIAs.

**Methods:**

We fitted interrupted time-series regressions to surveillance data between January 2016 and November 2023 to estimate the impact of nOPV2 and mOPV2 SIAs on cVDPV2 poliomyelitis incidence and prevalence in environmental surveillance across 37 countries, directly comparing the impact of SIAs in 13 countries where both vaccines were used.

**Results:**

We did not find any statistically significant differences between nOPV2 and mOPV2 SIA impact except for in the Democratic Republic of Congo (DRC), where nOPV2 SIAs had lower impact (adjusted relative risk [aRR] for cVDPV2 poliomyelitis incidence per nOPV2 SIA, 0.505; 95% confidence interval [CI], .409–.623) compared to mOPV2 (aRR, 0.193; 95% CI, .137–.272); *P* value for difference in RRs = 3e-6.

**Conclusions:**

We find variation in OPV2 SIA impacts globally, with greater certainty about Nigeria and DRC, where large outbreaks provided an opportunity to assess impact at scale. In most countries, we find no significant difference between nOPV2 and mOPV2 SIA impact. We are unable to identify the reason for the significant difference in DRC, which could include differential SIA coverage, timing, vaccine effectiveness, or outbreak dynamics.

## Teaser

Interrupted time-series analysis of surveillance data across 37 countries finds no significant difference between type-2 novel oral poliovirus vaccine and Sabin oral poliovirus vaccine on the incidence and prevalence of circulating vaccine-derived poliovirus, except in the Democratic Republic of Congo.

The Global Polio Eradication Initiative (GPEI) owes much of its success to the use of live-attenuated Sabin oral polio vaccine (OPV), which induces a strong mucosal immune response, is inexpensive, and easily administered compared to inactivated polio vaccine (IPV) [1]. However, OPV presents a critical challenge in completing eradication of all forms of polioviruses: the live vaccine virus may transmit from one susceptible individual to another. In communities where population immunity is persistently low, the virus may circulate for long enough to lose its attenuating mutations, regaining pathogenicity like that of wild poliovirus and causing outbreaks of what is termed circulating vaccine-derived poliovirus (cVDPV) [2, 3]. OPV can also regain pathogenicity by mutating rapidly in a single individual, termed vaccine-associated paralytic polio (VAPP) [4]. To reduce the risk of cVDPV and VAPP, type 2 OPV (OPV2) was withdrawn from routine immunization in 2016 as wild-type 2 poliovirus had been eradicated and the majority of cVDPVs detected were type 2. Unfortunately, postwithdrawal outbreaks of cVDPV2 required responses using monovalent OPV2 (mOPV2), leading to over 70 new emergences of cVDPV2 and cycles of outbreak and response [5, 6]. Between 1 May 2016 and 30 December 2023, 3253 cVDPV2 cases were reported [7].

Novel type 2 OPV (nOPV2) is based on the existing effective mOPV2 but designed with greater genetic stability to reduce the likelihood of future VDPV outbreaks [8]. nOPV2 showed good immunogenicity in clinical trials [9–11] and was granted emergency use listing by the World Health Organization (WHO) in 2020. nOPV2 was administered across 35 countries from March 2021 up to end December 2023, when nOPV2 was granted WHO prequalification [7]. nOPV2 has been used in response to explosive and persistent cVDPV2 outbreaks in Nigeria and Democratic Republic of Congo (DRC), with regions of Nigeria reporting uninterrupted transmission despite conducting as many as 10 SIAs in 24 months [12].

This analysis estimates the community-level impact of SIAs with nOPV2 and mOPV2 on the incidence of cVDPV2 poliomyelitis measured through surveillance for acute flaccid paralysis (AFP) and prevalence of cVDPV2 in environmental surveillance (ES) since withdrawal of trivalent OPV (tOPV) from routine immunization in April to May 2016. Because OPV effectiveness and SIA coverage tend to vary by setting [13, 14], we compare mOPV2 and nOPV2 SIA impact in similar contexts by estimating impact on multiple spatial scales, directly comparing SIAs with different vaccines in the same country or subnational region. Our analysis was planned prior to the first use of nOPV2 in an outbreak response to assess field performance under emergency use listing and contributed evidence in support of prequalification, with the primary objective of assessing the impact of nOPV2 SIAs relative to mOPV2 SIAs.

## METHODS

### Data

Poliomyelitis surveillance is conducted through AFP case notification and ES, with AFP cases listed by date of paralysis onset and residence (first and second administrative region, hereafter province and district, respectively) and ES samples by date of collection and identity and location of the sampling site [15, 16]. cVDPV2 poliomyelitis cases and environmental detections are identified through culture and sequencing of poliovirus in AFP or AFP contacts’ stool and sewage samples, respectively [17].

The GPEI maintains a calendar of polio SIAs including district-level information on implementation dates and vaccine formulation. Lot quality assurance sampling (LQAS) is a cluster survey method designed to detect whether coverage has likely exceeded (pass) or not exceeded (fail) a fixed threshold of 90% [18]. LQAS is conducted by a team independent from the vaccinator team and involves random sampling of 1 child (under 5 years of age) from 10 houses each in 6 randomly selected settlements per district immediately after a campaign [18]. We obtained data for SIAs and LQAS implemented and AFP and ES samples with onset and collection, respectively, through the Polio Information System [6].

To account for differences in population immunity, we used estimates of the proportion of children with type 2 immunity from OPV from a cohort model that assumes 50% coverage of SIAs and accounts for withdrawal of OPV2 from routine immunization in May 2016 [19, 20].

All data used in this study were collected as part of routine polio surveillance activities [15, 16]. Caregivers provided verbal consent to participation in surveillance activities on behalf of their children with the understanding that data may be used for multiple purposes to assist in polio eradication efforts. Institutional ethics approval for this study was granted by the Imperial College Research Governance and Integrity Team (reference ID 21IC6996).

### Statistical Analysis

We designed an interrupted time-series regression analysis to estimate the per-SIA impact of mOPV2 and nOPV2 SIAs conducted since May 2016 on district-level incidence of cVDPV2 poliomyelitis (notified through AFP surveillance) and prevalence of cVDPV2 in ES. We allow 90 days for laboratory confirmation and reporting of cVDPV2 AFP cases and ES samples [21]. We consider cVDPV2 cases or ES detections up to 90 days before the first and 90 days after the last SIA, meaning we evaluate the impact of SIAs conducted between 16 October 2016 and 5 November 2023 using surveillance data between 1 January 2016 and 7 November 2023. To account for cohort effects (ie, immunized individuals aging out of the high transmission group and susceptible individuals being born), we separate clusters of district-level SIAs more than 180 days apart (hereafter strata), assuming that these periods are independent. We divide each stratum into discrete periods based on the timing of SIAs and offset the timing of each SIA by 28 days, effectively assuming 28 days for an SIA to impact population immunity (consistent with polio vaccine trials [9–11]). We include strata with at least 1 case or detection, at least 1 mOPV2 or nOPV2 SIA, and no tOPV SIAs.

In 11 of 37 countries, geographical subdivisions changed during the analysis period. We use the current divisions as reference and reassign cases, ES samples, and SIAs from old divisions to current divisions following methods described in the Supplementary Material page 4.

To estimate the impact of SIAs on incidence, we fit a conditional quasi-Poisson regression. Let $Y_{ij}$ be the observed number of cVDPV2 cases in district *j* and period $i,\;\mathrm{and}\;Y_{ij}∼Poisson(\lambda_{ij},k)$ where *k* is the overdispersion parameter. We model the incidence as


(1) $$log(\lambda_{ij})=\alpha_{j}+b_{ij}\beta_{mq}+c_{ij}\beta_{nq}+\phi_{j}(b_{ij}+c_{ij})\beta_{\phi }+E_{ij}$$

where $\alpha_{j}$ is the pre-SIA incidence in the district; $\beta_{mq}$ or $\;\beta_{nq}$ give the log-relative reduction in incidence following an SIA with mOPV2 or nOPV2 in country *q*; $b_{ij}$, and $c_{ij}$ give the cumulative number of mOPV2 and nOPV2 SIAs conducted in district *j* as of time period *i*; $E_{ij}$ is an offset for the log-number of aged under 5 years person-days in time period *i*; $\phi_{j}$ is the proportion of children 6–36 months of age with type 2 immunity from OPV in district *j* 30 days before the first SIA, using estimates from a monthly cohort model that assumes 50% coverage of SIAs [19, 20]; and $\beta_{\phi }$ adjusts SIA impact according to prior immunity $\phi_{j}$. $\phi_{j}$ is multiplied by the sum of cumulative mOPV2 and nOPV2 cases $\left(b_{ij}+c_{ij}\right)$ to adjust for prior immunity equally for both vaccines. We do not consider vaccine induced immunity in infants aged under 6 months as this cohort typically have maternal antibodies [10, 13]. We use national estimates from World Population Prospects for children aged under 5 years distributed across districts according to the relative distribution of rasterized aged under 5 years population estimates [22, 23].

Pre-SIA incidence $\alpha_{j}$ will vary between strata. To overcome this limitation, we use a conditional quasi-Poisson regression, which conditions on the total incidence in each stratum [24].

We fit a similar model to estimate the impact of OPV2 SIAs on the prevalence of cVDPV2 in ES, using a conditional logistic regression because we have a fixed number of environmental samples ($N_{ij}$) in each sampling site *j* and period *i* (Supplementary Material page 7).

As a secondary analysis, we investigate the influence of LQAS results on incidence in Nigeria, modelling incidence as


(2) $$\begin{array}{r} log(\lambda_{ij})=\alpha_{j}+b_{ij}\beta_{mq}+c_{ij}\beta_{nq}+\phi_{j}(b_{ij}+c_{ij})\beta_{\phi }+\sum d_{kij}\beta_{k}+E_{ij} \end{array}$$

where $d_{kij}$ is the cumulative number of campaigns with *k* LQAS result (pass, fail, missing data) in the period *i*, and $\beta_{k}$ adjusts SIA impact according to LQAS result k. We adjust for LQAS in Nigeria only, because it has most data (254 of 738 strata in the incidence analysis) and complete reporting of LQAS results (351 of 1856 [19%] missing).

We fit these regressions using the “gnm” package in R version 4.2.1 [25, 26], once with an interaction term *q* for each country and once with an interaction term *q* for subnational regions in Nigeria and DRC (see Supplementary Table 1 for assignment of provinces to regions), because these are populous countries with large cVDPV2 outbreaks. To compare model fit, we use Aikike's information criterion (AIC) for binomial and *F* test for quasi-Poisson regression [27]. We test models with and without country-specific effects. We test for the difference in 2 risk or odds ratios as detailed in Supplementary Material page 9.

We assess sensitivity of our findings to the following assumptions: (1) days between SIAs, (2) days before and after a set of SIAs, (3) days for an SIA to take effect, (4) log-linear relationship between incidence or prevalence and cumulative SIAs (Supplementary Material page 8), and (5) adjusting for immunity equally for both mOPV2 and nOPV2 SIAs (Supplementary Material page 6).

## RESULTS

### Scale of Incidence, Prevalence, and Outbreak Response

**Figure 1.**
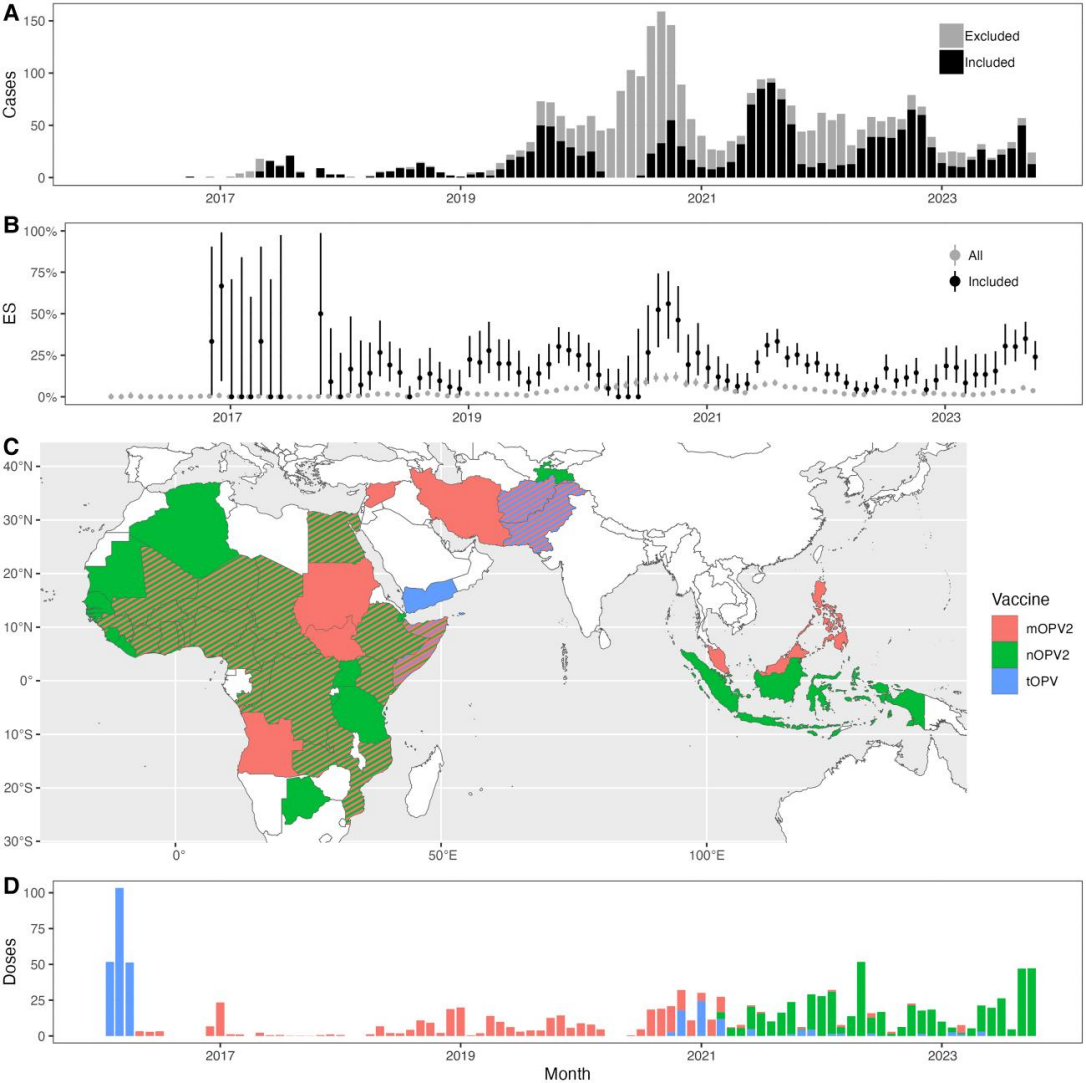
Summary of cVDPV2 incidence, prevalence, and outbreak response in World Health Organization African, Eastern Mediterranean, European, South-East Asian, and Western Pacific Regions. *A*, Monthly cVDPV2 poliomyelitis cases, 1 January 2016 to 1 November 2023. *B*, Monthly prevalence of cVDPV2 detection in environmental surveillance (error bars show 95% binomial confidence interval), 1 January 2016 to 1 November 2023. *C*, Map of countries implementing mOPV2, nOPV2, or tOPV SIAs, 1 May 2016 to 1 November 2023. *D*, Approximate number of monthly doses of mOPV2, nOPV2, and tOPV delivered in SIAs, 1 May 2016 to 1 November 2023 (estimated aged under 5 years population in targeted regions). Abbreviations: cVDPV2, circulating vaccine-derived type 2 poliomyelitis; mOPV2, monovalent type 2 oral poliovirus vaccine; nOPV2, novel type 2 oral poliovirus vaccine; SIA, supplementary immunization activities; tOPV, trivalent type 2 oral poliovirus vaccine.

In total, 3248 cases of cVDPV2 poliomyelitis were reported in WHO African, Eastern Mediterranean, European, South-East Asian, and Western Pacific Regions between 1 January 2016 and 1 November 2023, 1586 of which were within 90 days of a set of mOPV2 or nOPV2 SIAs (and no tOPV SIA), and therefore were included in this analysis (Figure 1*A*). Most cases occurred in Nigeria (593 total, 569 in analysis) and DRC (693 total, 464 in analysis). In the same period, 58 624 ES samples were collected from 1568 sites (1797 samples were positive for cVDPV2; Figure 1*B*); 5212 samples (903 positive) from 243 sites were included in this analysis.

Since 1 May 2016, mOPV2, nOPV2, and tOPV SIAs targeted an estimated 356, 525, and 84 million children aged under 5 years (Figure 1*C*). Seven countries have used only mOPV2, 14 countries have used only nOPV2, and 19 countries have used both vaccines (Figure 1*D*). Following our inclusion criteria, we analyzed outbreaks from 10 countries for mOPV2 impact, 11 countries for nOPV2 impact, and 16 countries for both mOPV2 and nOPV2 impact. Nigeria and DRC together account for 55% and 48% of the 743 and 307 strata included in the incidence and prevalence analysis, respectively (71% of nOPV2 strata; Supplementary Table 3 and Supplementary Figure 8).

### Global Variation in nOPV2 and mOPV2 SIA Impact

**Figure 2.**
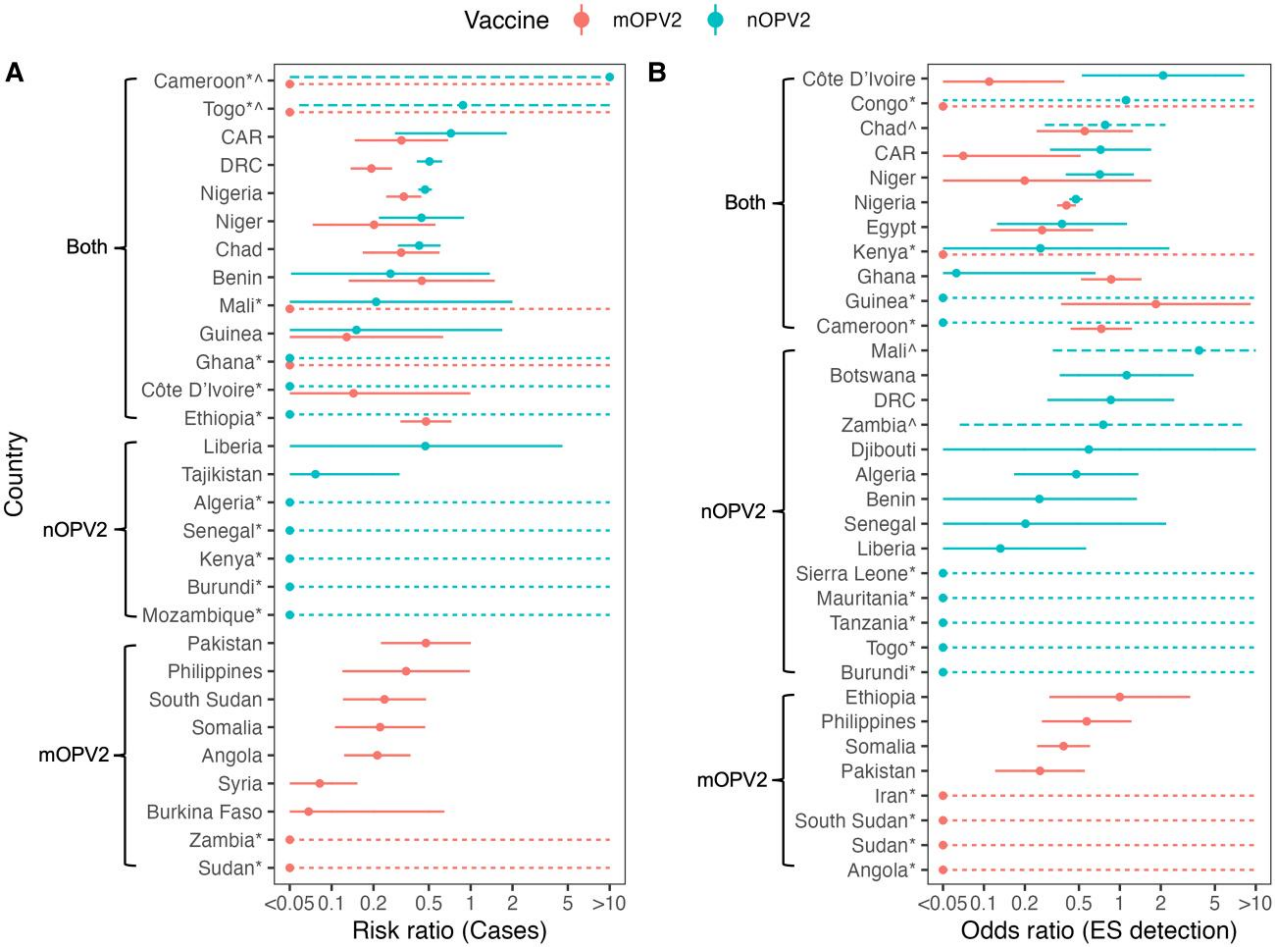
Log-linear per-SIA impact of mOPV2 and nOPV2 on (*A*) cVDPV2 poliomyelitis incidence (RR) and (*B*) prevalence in ES (OR), adjusting for immunity before the first SIA. Points show central RR or OR estimate and error bars show 95% CI. Countries are ordered from top to bottom by those included in both nOPV2 and mOPV2 analysis, nOPV2 only, and mOPV2 only, and then by magnitude of RR or OR central estimate. Countries with an asterisk (*) and dotted error bars have zero cases or detections following SIAs. Countries with caret (*^*) and dashed error bars have zero cases or detections before an SIA. Role of immunity: RR, 1.17 (95% CI, 1.12–1.21); OR, 1.12 (95% CI, 1.10–1.14) per 10% absolute increase. See Supplementary Tables 7 and 8 for a tabular representation of these results. Abbreviations: CAR, Central African Republic; CI, confidence interval; cVDPV2, circulating vaccine-derived type 2 poliomyelitis; DRC, Democratic Republic of Congo; ES, environmental surveillance; mOPV2, monovalent type 2 oral poliovirus vaccine; nOPV2, novel type 2 oral poliovirus vaccine; OR, odds ratio; RR, risk ratio; SIA, supplementary immunization activities.

We find that models with country-specific effects fitted significantly better than those without (*F* test for incidence model *P* value < .01, AIC for prevalence model 2800 vs 2693), indicating significant variability in mOPV2 and nOPV2 SIA impact between countries (Figure 2). mOPV2 SIAs had a significantly greater impact on incidence in Syria (adjusted risk ratio [aRR], 0.0821; 95% confidence interval [CI], .0440–.153) than in Ethiopia (aRR, 0.477; 95% CI, .312–.729; *P* value = 5e-6) or Nigeria (aRR, 0.330; 95% CI, .247–.442; *P* value = 8e-5). We find no other statistically significant pairwise differences between countries.

### Comparing nOPV2 and mOPV2 SIA Impact

We find no statistically significant difference between mOPV2 and nOPV2 SIA impact in any country except DRC, where we see a lower impact of nOPV2 SIAs compared to mOPV2 (aRR nOPV2, 0.505 [95% CI, .409–.623] vs aRR mOPV2, 0.193 [95% CI, .137–.272]; *P* value = 3e-6). Of 13 countries for which we have results for both nOPV2 and mOPV2 SIA impact, we find lower point estimates for nOPV2 SIA impact on incidence compared to mOPV2 in 9 countries (Cameroon, Central African Republic [CAR], DRC, Nigeria, Niger, Chad, Mali, Guinea, and Togo), higher point estimates for nOPV2 in 3 countries (Benin, Cote d’Ivoire, and Ethiopia), and no difference in 1 country (Ghana) (Figure 2*A*).

We find no statistically significant difference between mOPV2 and nOPV2 impact on prevalence in ES in any country, with lower point estimates for impact of nOPV2 compared to mOPV2 in 8 of 11 countries (Cote d’Ivoire, Congo, Chad, CAR, Niger, Nigeria, Egypt, and Kenya), and higher impact of nOPV2 in 3 countries (Ghana, Guinea, and Cameroon) (Figure 2*B*).

### Subnational Variation in nOPV2 and mOPV2 SIA Impact

**Figure 3.**
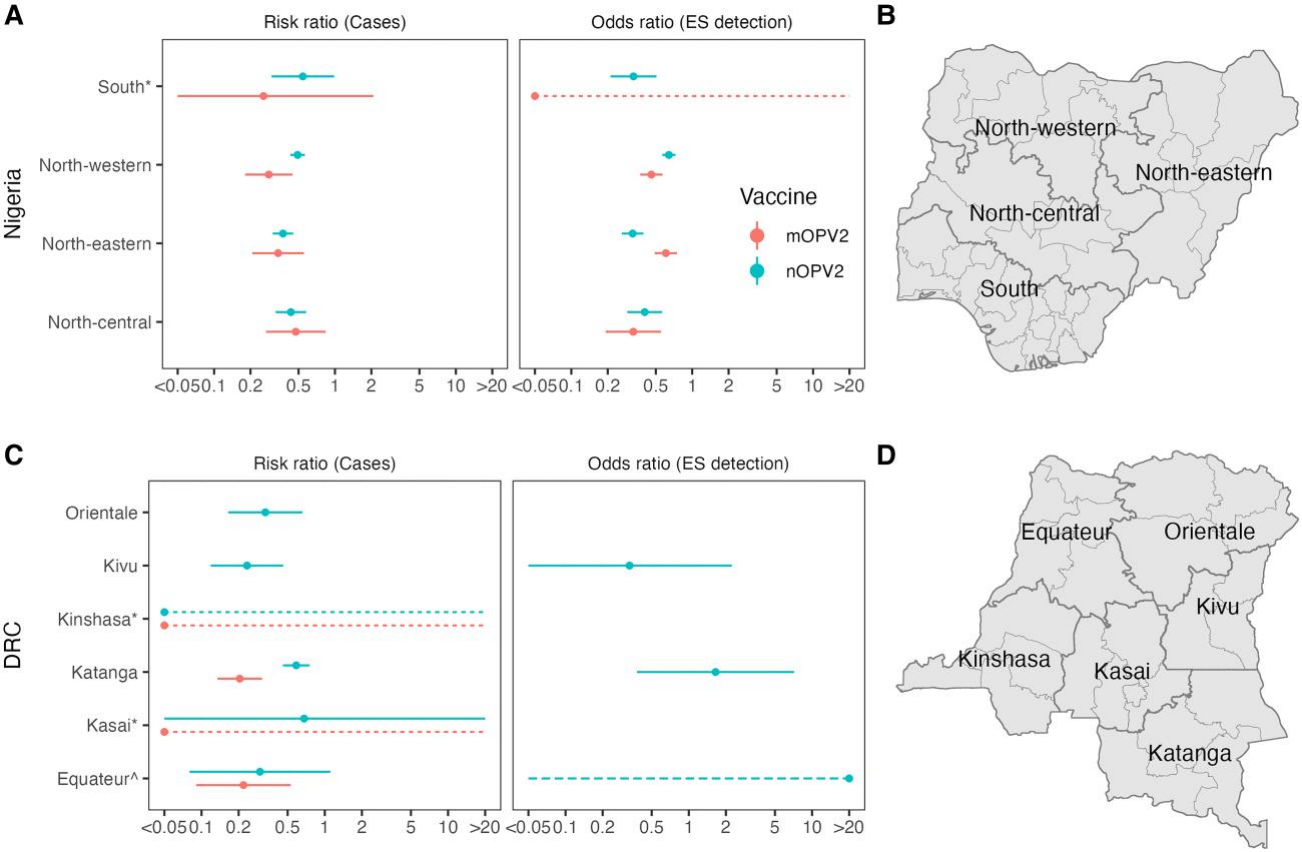
Log-linear per-SIA impact of mOPV2 and nOPV2 on cVDPV2 poliomyelitis incidence (RR) and prevalence in ES (OR) in Nigeria (*A*) and DRC (*C*) by subnational region (maps in (*B*) and (*D*), respectively), adjusting for immunity before the SIA. Points show central RR or OR estimate and vertical lines show 95% CI. Regions with an asterisk (*) and dotted error bars have zero cases or detections following SIAs. Regions with caret (*^*) and dashed error bars have zero cases or detections before an SIA. Role of immunity: RR, 1.17 (95% CI, 1.12–1.22); OR, 1.08 (95% CI, 1.06–1.11) per 10% absolute increase. See Supplementary Tables 9 and 10 for a tabular representation of these results. Abbreviations: CI, confidence interval; cVDPV2, circulating vaccine-derived type 2 poliomyelitis; DRC, Democratic Republic of Congo; ES, environmental surveillance; mOPV2, monovalent type 2 oral poliovirus vaccine; nOPV2, novel type 2 oral poliovirus vaccine; OR, odds ratio; RR, risk ratio; SIA, supplementary immunization activities.

Following our inclusion criteria, we compared the impact of nOPV2 and mOPV2 SIAs on (1) cVDPV2 incidence in all 4 subnational regions in Nigeria and 4 of 6 regions in DRC, and (2) prevalence in ES in all 4 regions of Nigeria and no regions of DRC. We only find a statistically significant differences in Katanga in DRC, where nOPV2 SIAs had a lower impact on incidence than mOPV2 (aRR nOPV2, 0.587 [95% CI, .457–.754] vs aRR mOPV2, 0.204 [95% CI, .134–.310]; *P* value = 2e-5; Figure 3); and in north-eastern Nigeria, where nOPV2 campaigns have had a greater impact than mOPV2 on prevalence (aOR nOPV2, 0.322 [95% CI, .262–.395] vs aOR mOPV2, 0.605 [95% CI, .489–.749]; *P* value = 3e-5).

### Role of Prior Immunity

Estimated type 2 immunity from OPV was higher in strata with nOPV2 use (n = 350; median 22%; interquartile range [IQR], 16%–35%) than in those with mOPV2 use (n = 312; median 0.6%; IQR, 0.4%–7.7%). Including vaccine-induced immunity significantly improved the fit for both incidence and prevalence country-level and subnational analyses (Supplementary Table 4), whereby SIAs in districts with higher vaccine-induced immunity in the month prior to the first campaign had a smaller impact (Figure 2, Figure 3, and Supplementary Tables 2–6).

### Role of LQAS

**Figure 4.**
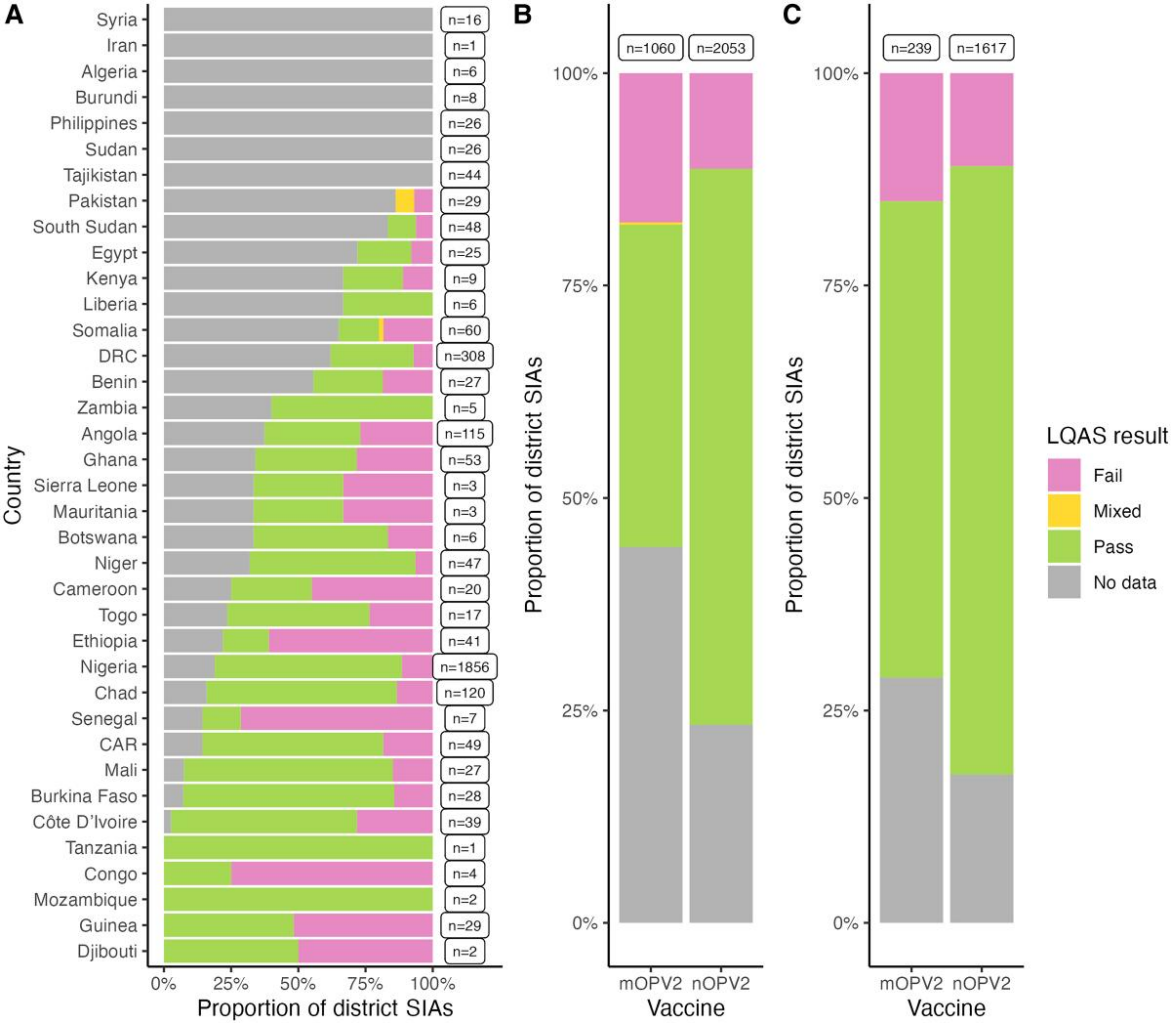
District-level LQAS results for nOPV2 and mOPV2 district SIAs included in interrupted time-series analysis (9 May 2016 to 1 November 2023) by country (*A*), by vaccine (*B*), and by vaccine in Nigeria (*C*). LQAS is a standardized cluster survey method designed to detect whether coverage at the district level has likely exceeded (pass) or not exceeded (fail) a fixed threshold of 90%. Where multiple clusters of settlements are sampled and 1 or more passes and 1 or more fails, this is defined as a mixed result. Abbreviations: CAR, Central African Republic; DRC, Democratic Republic of Congo; LQAS, lot quality assurance sampling; mOPV2, monovalent type 2 oral poliovirus vaccine; nOPV2, novel type 2 oral poliovirus vaccine; SIA, supplementary immunization activities.

In Nigeria, 1292 of 1856 district SIAs passed, 213 failed, and 351 were missing data (Figure 4), with a significantly higher pass rate for nOPV2 SIAs (*P* value < .01) and a significantly lower proportion of nOPV2 SIAs missing data (*P* value < .01). Including the LQAS result improved the model fit for incidence of poliomyelitis in Nigeria (*F* test *P* value = .01). There was a statistically significant difference between SIAs passing LQAS and those failing, and between SIAs that passed and SIAs for which no LQAS results were available (Table 1). Including LQAS also improved the model fit for prevalence in ES (AIC 1737 vs 1677), with SIAs failing LQAS or with missing data having significantly lower impact on prevalence than SIAs passing LQAS (Table 1).

**Table 1.** Log-Linear Per-SIA Impact of mOPV2 and nOPV2 on cVDPV2 Poliomyelitis Incidence (RR) and Prevalence in Environmental Surveillance (OR) in Nigeria, Adjusting for Immunity Before the SIA and LQAS Result

Factor	Impact on cVDPV2 Poliomyelitis Incidence				Impact on cVDPV2 Prevalence in Environmental Surveillance			
	Without Adjustment for LQAS Result		With Adjustment for LQAS Result		Without Adjustment for LQAS Result		With Adjustment for LQAS Result	
	aRR	(95% CI)	aRR	(95% CI)	aOR	(95% CI)	aOR	(95% CI)
mOPV2 SIA	0.353	(.259–.482)	0.266	(.193–.367)	0.402	(.343–.472)	0.262	(.213–.323)
nOPV2 SIA	0.493	(.432–.562)	0.406	(.347–.475)	0.473	(.422–.529)	0.355	(.304–.416)
Immunity, per 10% absolute increase	1.15	(1.10–1.20)	1.15	(1.10–1.20)	1.12	(1.10–1.14)	1.13	(1.11–1.16)
LQAS result								
Pass, likely > 90% coverage	…	…	CV	CV	…	…	CV	CV
Fail, likely < 90% coverage	…	…	1.61	(1.17–2.22)	…	…	1.54	(1.17–2.05)
No data	…	…	1.73	(1.35–2.23)	…	…	2.90	(2.09–4.03)
nOPV2 vs mOPV2								
Ratio of aRR and aOR	1.42		1.53		1.17		1.35	
*P* value for difference in aRR and aOR	.0522		.0206		.107		.0215	

LQAS is a standardized cluster survey method designed to detect whether coverage at the district level has likely exceeded (pass) or not exceeded (fail) a fixed threshold of 90%.

Abbreviations: aOR, adjusted odds ratio; aRR, adjusted risk ratio; CI, confidence interval; cVDPV2, circulating vaccine-derived type 2 poliomyelitis; CV, constrained variable; LQAS, lot quality assurance sampling; mOPV2, monovalent type 2 oral poliovirus vaccine; nOPV2, novel type 2 oral poliovirus vaccine; SIA, supplementary immunization activities.

Adjusting for LQAS result in Nigeria increased the difference between nOPV2 and mOPV2 SIA impact (ratio of nOPV2 to mOPV2 aRR with LQAS 1.53 vs 1.42 without LQAS, ratio of aOR 1.35 vs 1.17; *P* value = .0206 and .0215, respectively). This result was driven by data from north-western Nigeria (Supplementary Tables 5 and 6).

## DISCUSSION

This analysis examined incidence and prevalence of cVDPV2 in 37 countries to assess whether the impact of nOPV2 campaigns during the emergency use listing period was comparable to that of previous mOPV2 campaigns. We find wide variation in impact of mOPV2 and nOPV2 SIAs across geographies but no statistically significant differences between SIAs with the 2 vaccines in the same geographies apart from 1 country at the national level, DRC, where we find significantly lower impact of nOPV2 on cVDPV2 incidence relative to mOPV2. Adjusting for campaign quality using LQAS results in Nigeria resulted in a small but statistically significant difference between nOPV2 and mOPV2 SIA impact, with mOPV2 impact being greater.

The difference between SIA impact in DRC can be localized to the south-eastern provinces, where cVDPV2 incidence decreased significantly more following mOPV2 than nOPV2 SIAs. This observed difference may be due to differences in individual-level vaccine effectiveness (eg, findings of reduced immunogenicity of nOPV2 in young children when coadministered with bivalent OPV suggest that nOPV2 may be less robust than mOPV2 to competition with other viruses in the gut [28]), campaign coverage, campaign timing, changes in surveillance quality, secondary vaccine spread, outbreak dynamics that violate our methodological assumptions, or a combination of factors. It is difficult, if not impossible, to isolate each of these elements individually. Our results assess the impact of SIAs in different time periods with different vaccines rather than directly comparing individual-level effectiveness of vaccines. We were unable to adjust for coverage in DRC because of the limited availability of LQAS data. It is interesting to note that 4 new cVDPV2 lineages linked to nOPV2 were first detected in DRC [29]. Low prior immunity is a known risk factor for VDPV emergence [3], and poor campaign coverage may be a risk factor for emergence [30].

We find a significant correlation between SIA impact and LQAS results in Nigeria, where failing LQAS and reporting no LQAS results both predict lower impact. This finding is consistent with the observation that LQAS tends not to be performed in areas with poor accessibility or insecurity—the same areas where it is difficult to effectively deliver OPV. However, adjusting for LQAS does not explain the apparent differences between impact of nOPV2 and mOPV2 SIAs in Nigeria—the difference between nOPV2 and mOPV2 was greater after adjusting for LQAS result. Unfortunately, we are unable to assess the role of LQAS more broadly because many results are not collected in the Polio Information System.

The interrupted time-series analysis compares average incidence and prevalence over periods of time. Trends in incidence and prevalence may confound vaccination impact and may occur due to the size of the susceptible population, importation of new infections, and stochastic effects. However, it is not always possible to fit a mechanistic model that takes such complex factors into account when an outbreak is sparsely observed, where as few as 1 in 1900 poliovirus infections may result in paralysis [31], or where heterogeneity in population susceptibility and mixing is not well known. Previous work by Voorman and colleagues fitted a semimechanistic model to data from Nigeria, where cVDPV2 cases have been numerous [32].

We find that SIAs have a greater impact in populations with lower prior vaccine-induced immunity. This may be because our method measures the relative reduction in incidence or prevalence and outbreaks that start in populations with low immunity tend to grow faster [33], resulting in high incidence prior to vaccination and enabling a larger decrease in incidence to be measured.

This study depends on accurate reporting of SIA timing. Although the GPEI maintains a comprehensive centralized database of polio SIAs, there may be errors if postponed or cancelled SIAs are not reported. Our analysis also does not account for seasonality or naturally acquired immunity, both factors that we expect to have a minimal impact on outbreak dynamics in nonendemic settings where the major component of population immunity is vaccine induced [32].

This study finds few locations with a significant difference between nOPV2 and mOPV2 SIA impact, consistent with 2 other studies which found no significant difference between the 2 vaccines in Nigeria, although all 3 studies measured different aspects of vaccination impact. Voorman and colleagues estimated that nOPV2 and mOPV2 SIAs reduced the susceptible population in Nigeria by 42% (95% CI, 28%–54%) and 38% (95% CI, 20%–51%) on average, respectively [32]. A matched case-control study in Nigeria estimated a per-dose effectiveness against cVDPV2 poliomyelitis of 12% (95% CI, −2% to 25%) and 17% (95% CI, 3%–29%) for nOPV2 and mOPV2, respectively [12]. This analysis finds a per-SIA reduction in incidence of 53% (95% CI, 47%–58%) and 67% (95% CI, 56%–75%) for nOPV2 and mOPV2, respectively, in Nigeria.

Two previous studies used a similar framework to examine the impact of tOPV and tOPV-IPV combined campaigns on cVDPV2 and WPV1 incidence and prevalence in Nigeria and Pakistan, respectively [33, 34]. We did not consider the impact of outbreak response campaigns with IPV as these are limited to 10 countries. Previous modelling estimated a smaller and more uncertain impact of IPV campaigns relative to mOPV2 and nOPV2 in Nigeria between 2016 and 2022, possibly because IPV was used less frequently and in close succession with OPV, meaning that it was difficult to distinguish its contribution from that of OPV [32]. We also did not assess the impact of tOPV SIAs because these were relatively few in the study period [6] and its impact could not be combined with mOPV2, a monovalent vaccine with a different formulation and immunological effects [13].

Current standard operating procedures recommend completing 2 SIAs within 56 days of declaring a polio outbreak followed by an additional “mop-up”—targeted delivery in areas where campaign monitoring indicates low coverage—and further large-scale responses if additional cases or environmental detections occur after mop-up [35]. However, as case ascertainment and sample processing take time [21], 2 months may pass before an emergency operations center becomes aware of breakthrough transmission, further delaying response to an active outbreak. In settings where transmission is routinely detected after 2 SIAs, it may be beneficial to plan and conduct 3 SIAs in close succession.

We find substantial variation in impact of OPV2 SIAs globally, with greater certainty about relatively poor impact in Nigeria and DRC, where large outbreaks have provided an opportunity to assess performance at scale. In most locations, we find no significant difference in impact of nOPV2 and mOPV2 SIAs, suggesting that nOPV2 may continue to be used effectively in outbreak responses considering its enhanced genetic stability compared to mOPV2, which has been demonstrated in clinical studies and following large-scale field use [36, 37]. However, we do find significantly lower impact of nOPV2 SIAs relative to mOPV2 SIAs in DRC, the country with the second-most data in our analysis. There is a continued need to monitor the performance of nOPV2 in outbreak response and the emergence of nOPV2-derived cVDPV2 closely.

## Supplementary Data

Supplementary materials are available at *The Journal of Infectious Diseases* online (http://jid.oxfordjournals.org/). Supplementary materials consist of data provided by the author that are published to benefit the reader. The posted materials are not copyedited. The contents of all supplementary data are the sole responsibility of the authors. Questions or messages regarding errors should be addressed to the author.

## Supplementary Material

jiae614_Supplementary_Data

## Notes

***Acknowledgment***. We acknowledge the essential contributions of all polio surveillance officers to the successful implementation of this study.

***Author contributions***. L. V. C., I. M. B., N. C. G., A. S. B., and A. V. contributed to study concept and design. L. V. C., I. M. B., A. V., E. J. G., and N. C. G. developed the statistical methods. L. V. C. and I. M. B. accessed and verified all the data, and drafted the original report. L. V. C. performed the statistical analysis. L. V. C., I. M. B., and N. C. G. interpreted the findings of the statistical analysis. All authors contributed to critical revision of the manuscript for intellectual content.

***Data availability.*** The detailed disease surveillance data on which this research is based are not available outside partners of the Global Polio Eradication Initiative.

***Financial support***. This work was supported by the Bill and Melinda Gates Foundation (grant number INV-031605) and the World Health Organization (grant number 2022/1217583–0 to I. M. B.). I. M. B. and N. C. G. acknowledge funding from the MRC Centre for Global Infectious Disease Analysis (grant number MR/X020258/1), funded by the UK Medical Research Council (MRC). This UK funded award is carried out in the frame of the Global Health EDCTP3 Joint Undertaking.
