# Supplementary material for: Global Impact of Mass Vaccination Campaigns on Circulating Type 2 Vaccine-Derived Poliovirus Outbreaks: An Interrupted Time-Series Analysis
Source: J Infect Dis. 2025 Jan 28;231(2):e446–55. doi: 10.1093/infdis/jiae614 (PMC11841638; doi:10.1093/infdis/jiae614)
Supplement: jiae614_Supplementary_Data [file jiae614_supplementary_data.pdf]

Appendix to *Global impact of mass vaccination campaigns on circulating type 2 vaccine-derived poliovirus outbreaks: an interrupted time-series analysis*

Laura V Cooper[1*], Ananda S Bandyopadhyay[2], Nicholas C Grassly[1], Elizabeth J Gray[1], Arie Voorman[2], Simona Zipursky[2], Isobel M Blake[1]

1. MRC Centre for Global Infectious Disease Analysis, School of Public Health, Imperial College London, London, United Kingdom
2. Bill & Melinda Gates Foundation, Seattle, United States of America

*Corresponding author: l.cooper@imperial.ac.uk

Table of Contents

SUPPLEMENTARY METHODS 3

Figure S1. Visual representation of interrupted time-series in (a) Mayadeen, Syria, and (b) Kongolo, DRC. Grey bars show weekly cVDPV2 cases. Yellow arrows show the timing of implementation of SIAs. Green lines show the timing of the SIAs taking effect on population immunity (28 days later). Red and blue lines show the start and end of the time-series analysis (90 days before and after the first and last SIA). Purple line shows the mean weekly incidence of cases in each discrete period. 4

Figure S2. Translation of old shapes to new shapes, including LQAS data, for SIAs included in analysis. 5

Table S1. Aggregation of first-level administrative regions of DRC and Nigeria into larger sub-national regions. 9

Table S2. Number of pairwise tests for difference of two risk ratios and odds ratios and Bonferroni-adjusted p-value cut-off assuming family error rate of 0.05 by model. Number of tests for comparing regions using same vaccines calculated as total possible combinations of two (C2n). 10

Figure S3. Sensitivity analyses for log-linear per-SIA impact of mOPV2 (circle) and nOPV2 (cross) on cVDPV2 incidence (risk ratio [RR]) and prevalence in environmental surveillance (odds ratio [OR]), adjusting for immunity before the first SIA. 12

Figure S4. Sensitivity analyses for log-linear per-SIA impact of mOPV2 (circle) and nOPV2 (cross) on cVDPV2 incidence (risk ratio, RR) and prevalence in environmental surveillance (odds ratio, OR) in DRC and Nigeria by sub-national region, adjusting for immunity before the SIA. Points show central RR or OR estimate and vertical lines show 95% confidence interval (CI). Regions with an asterisk (*) and dotted error bars have zero cases or detections following SIAs. Regions with caret (^) and dashed error bars have zero cases or detections before an SIA. 13

Figure S5. Cumulative impact of one, two, three or four or more mOPV2 or nOPV2 SIAs on cVDPV2 incidence (risk ratio, RR), adjusting for immunity before the SIA. Points show central RR estimate and vertical lines show 95% confidence interval (CI). Where no point is shown, there is no stratum with this number of cumulative SIAs. Points with dashed error bars have zero cases after the SIA. Role of immunity: RR 1.09 (95% CI 1.06, 1.12) per 10% absolute increase. 14

Figure S6. Cumulative impact of one, two, three or four or more mOPV2 or nOPV2 SIAs on cVDPV2 prevalence in environmental surveillance (odds ratio, OR), adjusting for immunity before the SIA. Points show central OR estimate and vertical lines show 95% confidence interval (CI). Where no point is shown, there is no stratum with this number of cumulative SIAs. Points with dashed error bars have zero detections before and/or after the SIA (distinguished by shape). Role of immunity: OR 1.06 (95% CI 1.04, 1.07) per 10% absolute increase. 15

Figure S7. Cumulative impact of one, two, three or four or more mOPV2 or nOPV2 SIAs on cVDPV2 incidence (risk ratio, RR) or prevalence in environmental surveillance (odds ratio, OR), adjusting for immunity before the SIA. Points show central RR or OR estimate and vertical lines show 95% confidence interval (CI). Where no point is shown, there is no stratum with this number of cumulative SIAs. Points with dashed error bars have zero detections before and/or after the SIA (distinguished by shape). Lines and shaded ribbons indicate central estimate and 95% CI of log-linear model. 16

SUPPLEMENTARY RESULTS 17

Table S3. Number of strata included in incidence and prevalence analyses by country and vaccine type. 17

Table S4. Statistical tests for model fits with and without adjustment for population immunity. 18

Table S5. Log-linear per-SIA impact of mOPV2 and nOPV2 on cVDPV2 incidence (risk ratio, RR) across four regions of Nigeria, adjusting for immunity before the SIA and LQAS result. **Constrained variable. 19

Table S6. Log-linear per-SIA impact of mOPV2 and nOPV2 on cVDPV2 prevalence in ES (odds ratio, OR) across four regions of Nigeria, adjusting for immunity before the SIA and LQAS result. **Constrained variable. 19

Table S7. Log-linear per-SIA impact of mOPV2 and nOPV2 on cVDPV2 incidence, adjusting for immunity before the first SIA. Role of immunity: RR 1.17 (95% CI 1.12, 1.21) per 10% absolute increase. Immunity is mean district-level type 2 vaccine-induced immunity in children 6-36 months of age 30 days prior to first SIA. 20

Table S8. Log-linear per-SIA impact of mOPV2 and nOPV2 on cVDPV2 prevalence in ES, adjusting for immunity before the first SIA. Role of immunity: OR 1.12 (95% CI 1.10, 1.14) per 10% absolute increase. Immunity is mean district-level type 2 immunity in children 6-36 months of age 30 days prior to first SIA. 22

Table S9. Log-linear per-SIA impact of mOPV2 and nOPV2 on cVDPV2 incidence in sub-national regions of DRC and Nigeria, adjusting for immunity before the first SIA. Role of immunity: RR 1.17 (95% CI 1.12, 1.22) per 10% absolute increase. Immunity is mean district-level type 2 immunity in children 6-36 months of age 30 days prior to first SIA. 24

Table S10. Log-linear per-SIA impact of mOPV2 and nOPV2 on cVDPV2 prevalence in ES in sub-national regions of DRC and Nigeria, adjusting for immunity before the first SIA. Role of immunity: OR 1.08 (95% CI 1.06, 1.11) per 10% absolute increase. Immunity is mean district-level type 2 immunity in children 6-36 months of age 30 days prior to first SIA. 25

Table S11. Log-linear per-SIA impact of mOPV2 and nOPV2 on cVDPV2 incidence, adjusting for vaccine-induced population immunity before the first SIA separately for mOPV2 and nOPV2 SIAs. 26

Table S12. Log-linear per-SIA impact of mOPV2 and nOPV2 on cVDPV2 prevalence in environmental surveillance, adjusting for vaccine-induced population immunity before the first SIA separately for mOPV2 and nOPV2 SIAs. 27

Figure S8. Proportion of strata by analysis, vaccine, and country. Seven most frequent countries identified and less frequent countries aggregated into "Other". Total strata with mOPV2 or nOPV2 in incidence 28

Supplementary methods

We design an interrupted time-series regression analysis to estimate the per-SIA impact of mOPV2 and nOPV2 SIAs on district-level incidence and prevalence of cVDPV2 between 2016 and 2023. We allow 90 days for processing of AFP cases and ES samples, assuming our data set represents cVDPV2 incidence and prevalence between 1 January 2016 and 1 November 2023. To account for cohort effects (i.e. immunised individuals aging out of the high transmission group and susceptible individuals being born), we create separate clusters of district-level SIAs more than 180 days apart (hereafter strata), assuming that these periods are independent. We divide each stratum into discrete periods based on the timing of SIAs and offset the timing of each SIA by 28 days, effectively assuming 28 days for an SIA to impact population immunity (consistent with polio vaccine trials [9-11], see Figure S1). We consider cVDPV2 cases or ES detections up to 90 days before the first and 90 days after the last SIA in a stratum. We include strata with at least one case or detection, at least one mOPV2 or nOPV2 SIA, and no tOPV SIAs.

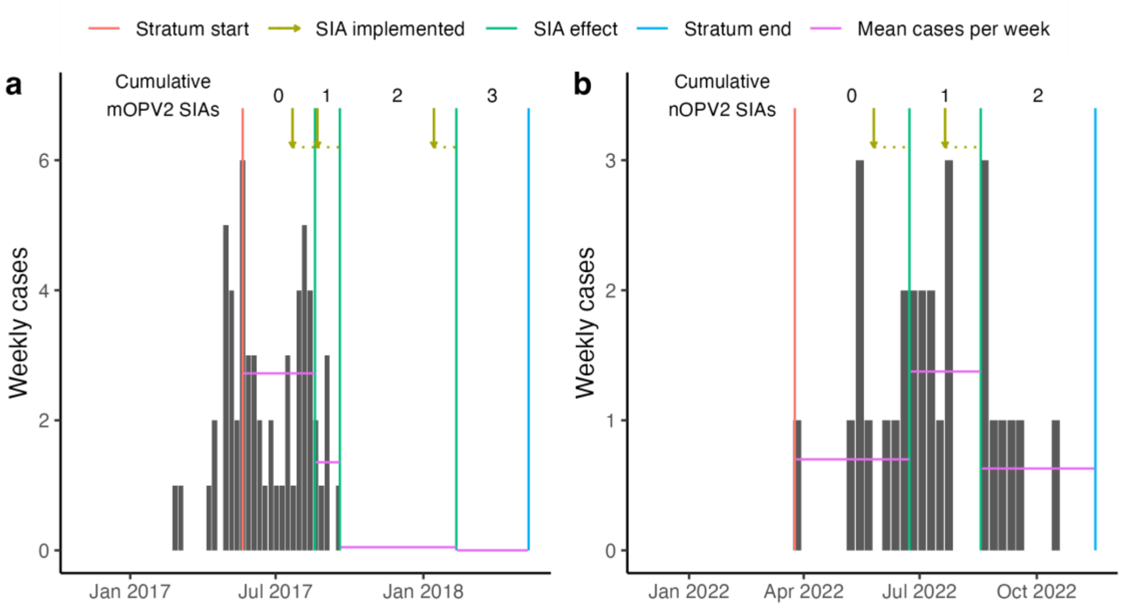

Figure S1. Visual representation of interrupted time-series in (a) Mayadeen, Syria, and (b) Kongolo, DRC. Grey bars show weekly cVDPV2 cases. Yellow arrows show the timing of implementation of SIAs. Green lines show the timing of the SIAs taking effect on population immunity (28 days later). Red and blue lines show the start and end of the time-series analysis (90 days before and after the first and last SIA). Purple line shows the mean weekly incidence of cases in each discrete period.

In 11 of 37 countries with OPV SIAs included in the analysis, geographical subdivisions have changed over the analysis period. We use the current divisions as our reference and assign cases and ES samples linked to old divisions by finding the current division which overlaps with a randomly assigned point within the old division or the exact geolocation of the ES site, where available. We assign SIAs linked to old divisions to current divisions if at least 50% of the current division's area is covered by the old division. Where current divisions are covered by SIAs linked to multiple old divisions, we assign LQAS results as a single result if more than 90% of the current division area is covered by a single type of result (pass, fail), or as mixed if less than 90% is covered by a single result (i.e. if 50% of the area is an old division which failed and 50% is an old division which passed). In assigning an LQAS result, we exclude old divisions which make up less than 5% of the current division

area, as these usually represent small variations in how boundaries are drawn from one map to the next rather than changes in real-world geography. Across the 43 countries where nOPV2 or mOPV2 have been used, we translate the old boundaries to new boundaries for 2564 district-SIAs, 2281 of which (89%) have a one-to-one correspondence with the old boundaries (Figure S2).

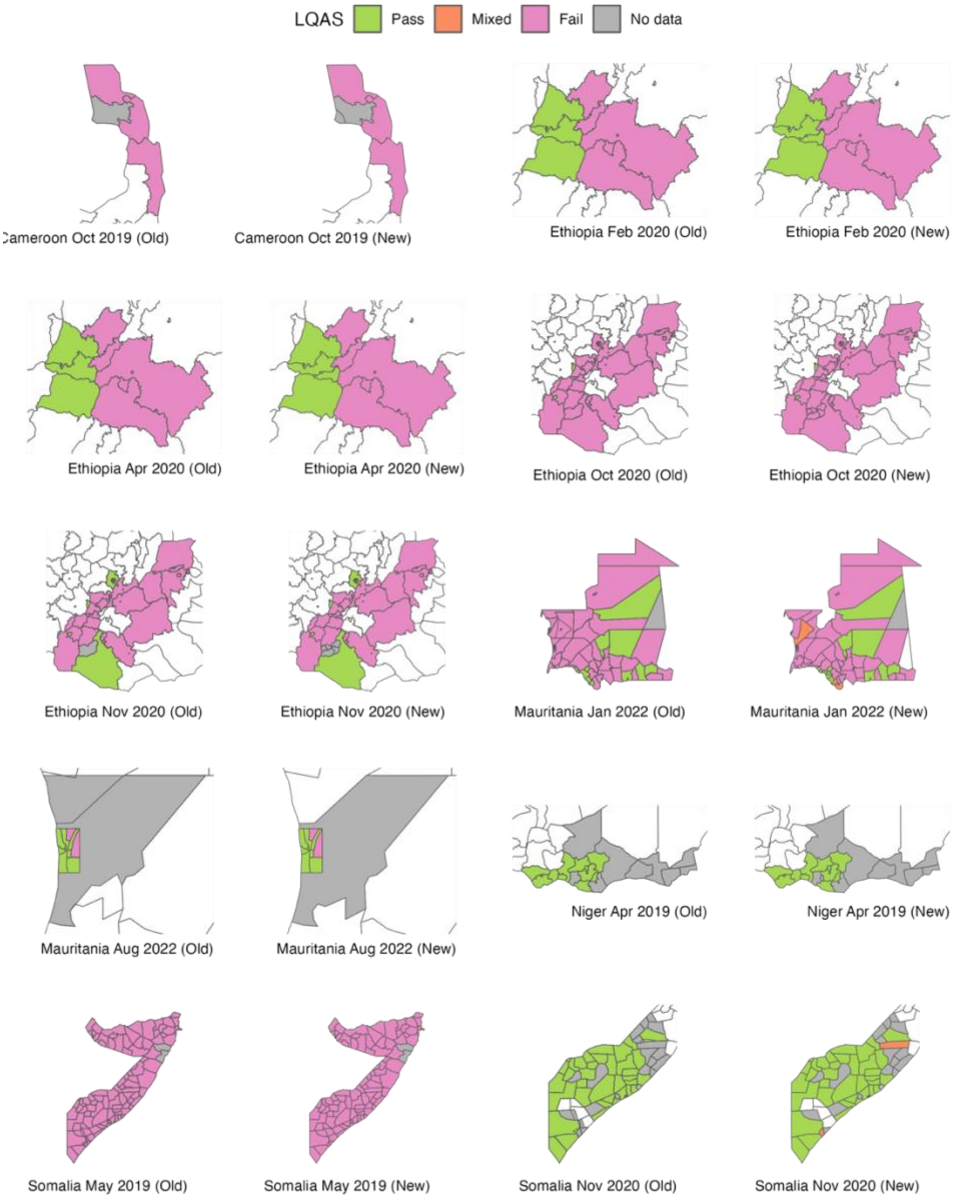

Figure S2. Translation of old shapes to new shapes, including LQAS data, for SIAs included in analysis.

To estimate the per-round impact of mOPV2 and nOPV2 SIAs on incidence of cases, we fit a conditional quasi-Poisson regression. Let Y_{ij} be the observed number of cVDPV2 cases in district j and time period i, and $Y_{ij} \sim Poisson(\lambda_{ij}, k)$ where k is the overdispersion parameter. We model the rate of disease as

$$log(\lambda_{i,j}) = \alpha_j + b_{ij}\,\beta_{mq} + c_{ij}\,\beta_{nq} + \phi_j(b_{ij} + c_{ij})\beta_\phi + E_{ij} \qquad (\mathbf{1})$$

where α_j is the pre-SIA rate of disease in the district, β_{mq} gives the log-relative reduction in disease following an SIA with mOPV2 in country q, b_{ij} gives the cumulative number of mOPV2 SIAs that had been conducted in district j as of time period i, β_{nq} gives the log-relative reduction in disease following an SIA with nOPV2 in country q, c_{ij} gives the cumulative number of nOPV2 SIAs that had been conducted in district j as of time period i, E_{ij} is an offset for the log-number of under-five person-days in time period i, ϕ_j is the proportion of children 6-36 months of age with type 2 immunity from OPV in district j 30 days before the first SIA, using estimates from a monthly cohort model which assumes 50% coverage of SIAs [1], and β_ϕ adjusts SIA impact according to prior immunity ϕ_j. ϕ_j is multiplied by the sum of cumulative mOPV2 and nOPV2 cases $(b_{ij} + c_{ij})$ in order to adjust for prior immunity equally for both vaccines. For example, let β_{nq}= -0.52, β_ϕ= 0.9, equivalent to a relative reduction in disease following one nOPV2 SIA of exp(-0.52) = 0.3 or 70% assuming ϕ_j=0, or a relative reduction of exp(-0.52 + 0.9[0.5]) = 0.93 or 7% assuming ϕ_j=0.5 (population with 50% immunity from OPV). In a sensitivity analysis, we allow for prior immunity to modulate the impact of nOPV2 and mOPV2 SIAs differently:

$$log(\lambda_{i,j}) = \alpha_j + b_{ij}\,\beta_{mq} + c_{ij}\,\beta_{nq} + \phi_j b_{ij}\beta_{m\phi} + \phi_j c_{ij}\beta_{n\phi} + E_{ij} \qquad (\mathbf{2})$$

We use national under-five population estimates from World Population Prospects distributed across districts according to the relative distribution of rasterized under-five population estimates [2,3]. As shown in equation (1), we assume a log-linear relationship between incidence and cumulative SIAs, but relax this assumption in a later sensitivity analysis.

The baseline rate of disease α_j will vary from district to district, as well as over time. Adjusting for all possible confounding factors would be problematic, if not impossible. To overcome this limitation we use a conditional quasi-poisson regression model, an extension of the conditional logistic regression model, which conditions on the total number of cases in each district [4]. In the simplest case, if there are Y cases in a district and one nOPV2 SIA, the null hypothesis (no effect of the SIA) would estimate equal probability that cases occurred before or after the SIA, whereas the alternative hypothesis would estimate the relative proportion of cases after the SIA would be $\exp(\beta_n)$. Over many districts one can estimate the average reduction in incidence following one SIA without having to estimate the underlying rate of disease in the area (α_j), since all that matters under the conditional model is the relative proportion of cases before or after the SIA [4].

We fit a similar model to environmental samples to estimate the impact of mOPV2 or nOPV2 SIAs on the prevalence of cVDPV2 in the environment (p_{ij}), using a conditional logistic regression instead because we have a fixed number of environmental samples (N_{ij}) in each environmental sampling site and time period. Let Z_{ij} be the observed number of cVDPV2 positive samples at site j and time period i and that $Z_{ij} \sim Binomial(p_{ij}, N_{ij})$. We model the prevalence of virus as

$$logit(p_{ij}) = \alpha_j + b_{ij}\,\beta_{m,q} + c_{ij}\,\beta_{nq} + \phi_j(b_{ij} + c_{ij})\beta_\phi \qquad \mathbf{(3)}$$

where α_j is the baseline prevalence at the site, β_{mq} gives the log-relative reduction in prevalence following an SIA with mOPV2 in country q, β_{nq} gives the log-relative reduction in prevalence following an SIA with nOPV2 in country q, and using the same definitions as above for b_{ij}, c_{ij}, β_ϕ, ϕ_j.

As a secondary analysis, we investigate the influence of LQAS results on incidence in Nigeria, modelling the rate of disease as

$$log(\lambda_{ij}) = \alpha_j + b_{ij}\,\beta_{m\,q} + c_{ij}\,\beta_{nq} + \phi_j(b_{ij} + c_{ij})\beta_\phi + \sum d_{kij}\,\beta_k + E_{ij} \qquad \textbf{(4)}$$

where d_{kij} is the cumulative number of campaigns with k LQAS result (pass, fail, mixed, invalid, missing data) in the time period i, and β_k adjusts SIA impact according to LQAS result k. We also test the influence of LQAS results on prevalence in ES as above.

The above models assume a log-linear relationship between incidence and cumulative SIAs. In a sensitivity analysis, we relax this assumption, allowing for independent effects of 1, 2, 3, or 4 or more cumulative SIAs:

$$log(\lambda_{ij}) = \alpha_j + \sum f_{ijr}\beta_{mrq} + \sum g_{ijs}\beta_{nsq} + \phi_j(b_{ij} + c_{ij})\beta_\phi + E_{ij} \qquad \textbf{(5)}$$

where f_{ijr} = 1 if the cumulative number of mOPV2 campaigns is equal to r and β_{mrq} gives the log-relative reduction in disease following the rth SIA with mOPV2 in country q (and f_{ijr} = 0 otherwise), and g_{ijs} = 1 if the cumulative number of nOPV2 campaigns is equal to s and β_{nsq} gives the log-relative reduction in disease following the sth SIA with nOPV2 in country q (and g_{ijs} = 0 otherwise).

We fit these regressions using the “gnm” package in R version 4.2.1 [5], once with an interaction term q for each country and once with an interaction term q for sub-national regions in Nigeria and DRC (see Table S1 for assignment of provinces to regions), because these are populous countries which experienced large cVDPV2 outbreaks. To compare

model fit, we use AIC for binomial and F-test for quasi-Poisson regression [6]. We allow the impact of OPV2 SIAs to vary between countries because models with country-specific effects fitted significantly better than models without country-specific effects (F-test for incidence model p-value < 0.01, AIC for prevalence model 2800 versus 2693).

Table S1. Aggregation of first-level administrative regions of DRC and Nigeria into larger sub-national regions.

Country	Sub-national region	Province
DRC	Equateur	Equateur, Mongala, Nord Ubangi, Sud Ubangi, Tshuapa
	Kasai	Kasai Central, Kasai Occidental, Kasai Oriental, Lomami, Sankuru
	Katanga	Haut Katanga, Haut Lomami, Katanga, Lualaba, Tanganyika
	Kinshasa	Bandundu, Bas Congo, Kinshasa, Kongo Central, Kwango, Kwilu, Maindombe
	Kivu	Maniema, Nord Kivu, Sud Kivu
	Orientale	Bas Uele, Haut Uele, Ituri, Orientale, Tshopo
Nigeria	North-central	Benue, FCT, Kogi, Kwara, Nasarawa, Niger, Plateau
	North-eastern	Adamawa, Bauchi, Borno, Gombe, Taraba, Yobe
	North-western	Jigawa, Kaduna, Kano, Katsina, Kebbi, Sokoto, Zamfara
	South	Abia, Akwa Ibom, Anambra, Bayelsa, Cross River, Delta, Ebonyi, Edo, Ekiti, Enugu, Imo, Lagos, Ogun, Ondo, Osun, Oyo, Rivers

We test for the difference in two risk or odds ratios by taking a p-value for the ratio $z = |x_1 - x_2| / \sqrt[2]{s_1 + s_2}$ from the standard normal, where x is the log-odds and s is the standard error. We use a Bonferroni correction with a family error rate of 0.05 and a number of tests equal to the number of pairwise comparisons in each regression to determine a p-value threshold for significance (Table S2).

We assess sensitivity of our findings to the following assumptions: i) days between SIAs (120 days versus baseline 180 days), ii) days before and after a set of SIAs (120 days versus baseline 90 days), iii) days for an SIA to take effect (0 days versus baseline 28 days), iv) log-linear relationship between incidence or prevalence and cumulative SIAs, v) adjusting for immunity equally for both mOPV2 and nOPV2 SIAs.

Table S2. Number of pairwise tests for difference of two risk ratios and odds ratios and Bonferroni-adjusted p-value cut-off assuming family error rate of 0.05 by model. Number of tests for comparing regions using same vaccines calculated as total possible combinations of two (C_2^n).

Model		Comparing regions using same vaccines				Comparing vaccines in same regions		Total tests	P-value cut-off
		mOPV2		nOPV2					
		Regions	Tests	Regions	Tests	Regions	Tests		
Country-level incidence		22	231	20	190	13	13	434	1.2e-4
Country-level prevalence		19	171	25	300	11	11	482	1.0e-4
Sub-national incidence	Nigeria	4	6	4	6	4	4		
	DRC	4	6	6	15	4	4		
	Total	8	12	10	21	8	8	41	1.2e-3
Sub-national prevalence	Nigeria	4	6	4	6	4	4		
	DRC	0	0	3	3	0	0		
	Total	4	6	7	9	4	4	19	2.6e-3

Sensitivity analyses

None of the sensitivity analyses changed our country-level findings with respect to differences between nOPV2 and mOPV2 impact in the same countries (Figure S3, S4). Our finding of significantly better impact of mOPV2 SIAs on incidence in Syria relative to Nigeria and Ethiopia was sensitive to our assumptions, where changing the number of days before or after SIAs from 90 to 120 (for Ethiopia), changing the number of days required to separate strata from 120 to 180 (for both countries), or assuming an immediate impact of SIAs (for Ethiopia) resulted in no significant difference between the countries. The differences between nOPV2 and mOPV2 impact identified with the log-linear model persist with the non-linear model: we find significantly poorer impact of nOPV2 on incidence relative to mOPV2 after 2 rounds in DRC (Figure S5), but no significant differences between vaccines in other countries or for impact on prevalence in ES (Figure S6). None of the sensitivity analyses significantly changed our sub-national findings (Figures S4, S7). We still find a statistically significant difference between nOPV2 and mOPV2 SIAs in Katanga in DRC, where nOPV2 SIAs had lower impact on incidence than mOPV2 (Figure S4a). We also still find a statistically significant difference in north-eastern Nigeria, where nOPV2 campaigns

have had a greater impact than mOPV2, and that nOPV2 SIAs had a significantly greater impact on prevalence in ES in north-eastern Nigeria than in north-western Nigeria (Figure S4b).

We also found no significant difference in the influence of prior immunity on nOPV2 and mOPV2 SIA impact on cVDPV2 incidence (RR for mOPV2 per 10% absolute increase in immunity 1.18 95% CI 1.08-1.29 versus for nOPV2 1.16 95% CI 1.12-1.21, Table S11) or prevalence in ES (OR for mOPV2 per 10% absolute increase in immunity 1.10 95% CI 1.04-1.16 versus for nOPV2 1.12 95% CI 1.10-1.15, Table S12).

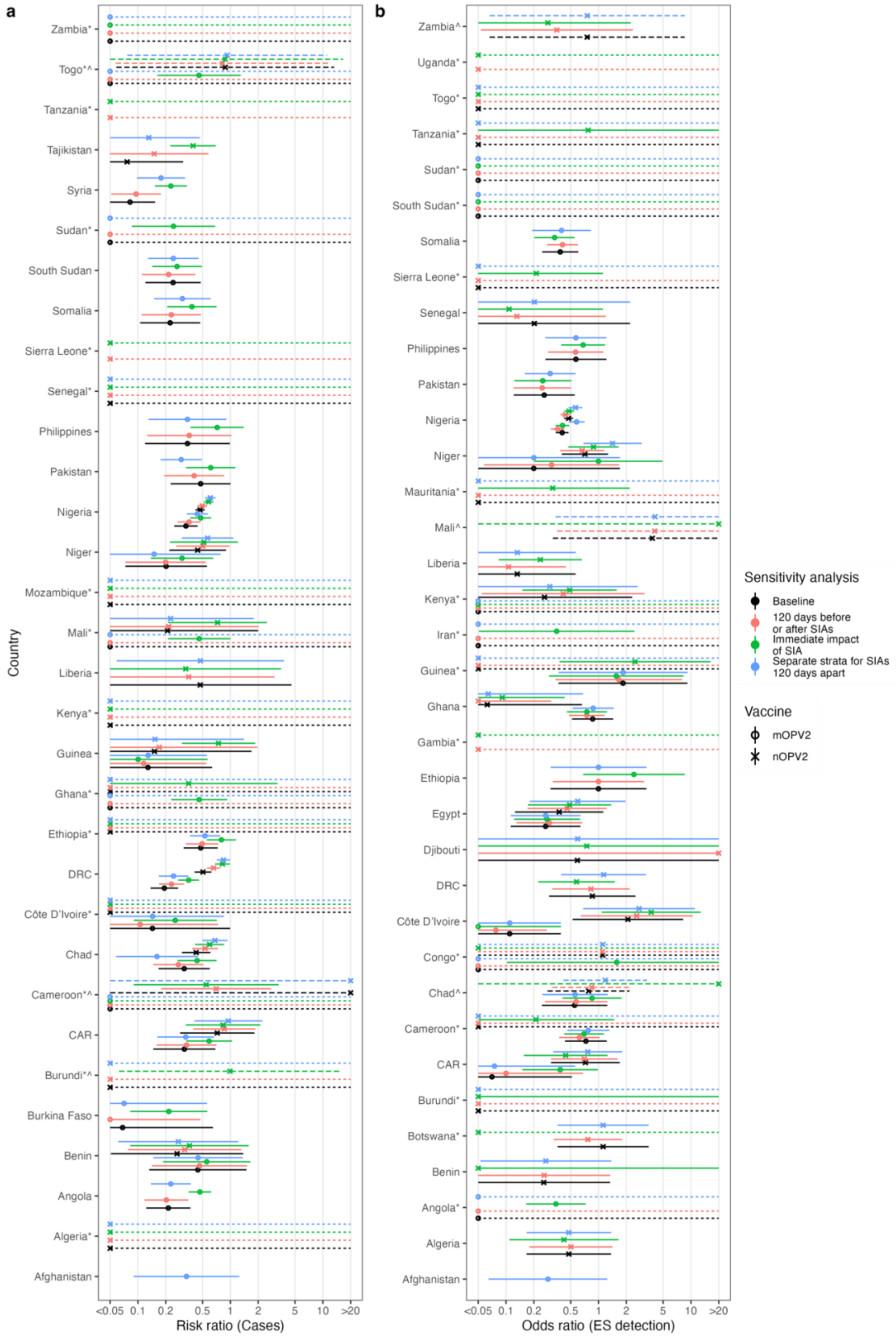

Figure S3. Sensitivity analyses for log-linear per-SIA impact of mOPV2 (circle) and nOPV2 (cross) on cVDPV2 incidence (risk ratio [RR]) and prevalence in environmental surveillance (odds ratio [OR]), adjusting for immunity before the first SIA. Points show central RR or OR estimate and vertical lines show 95% confidence interval (CI). Countries with an asterisk (*) and dotted error bars have zero cases or detections following SIAs. Countries with caret (^) and dashed error bars have zero cases or detections before an SIA.

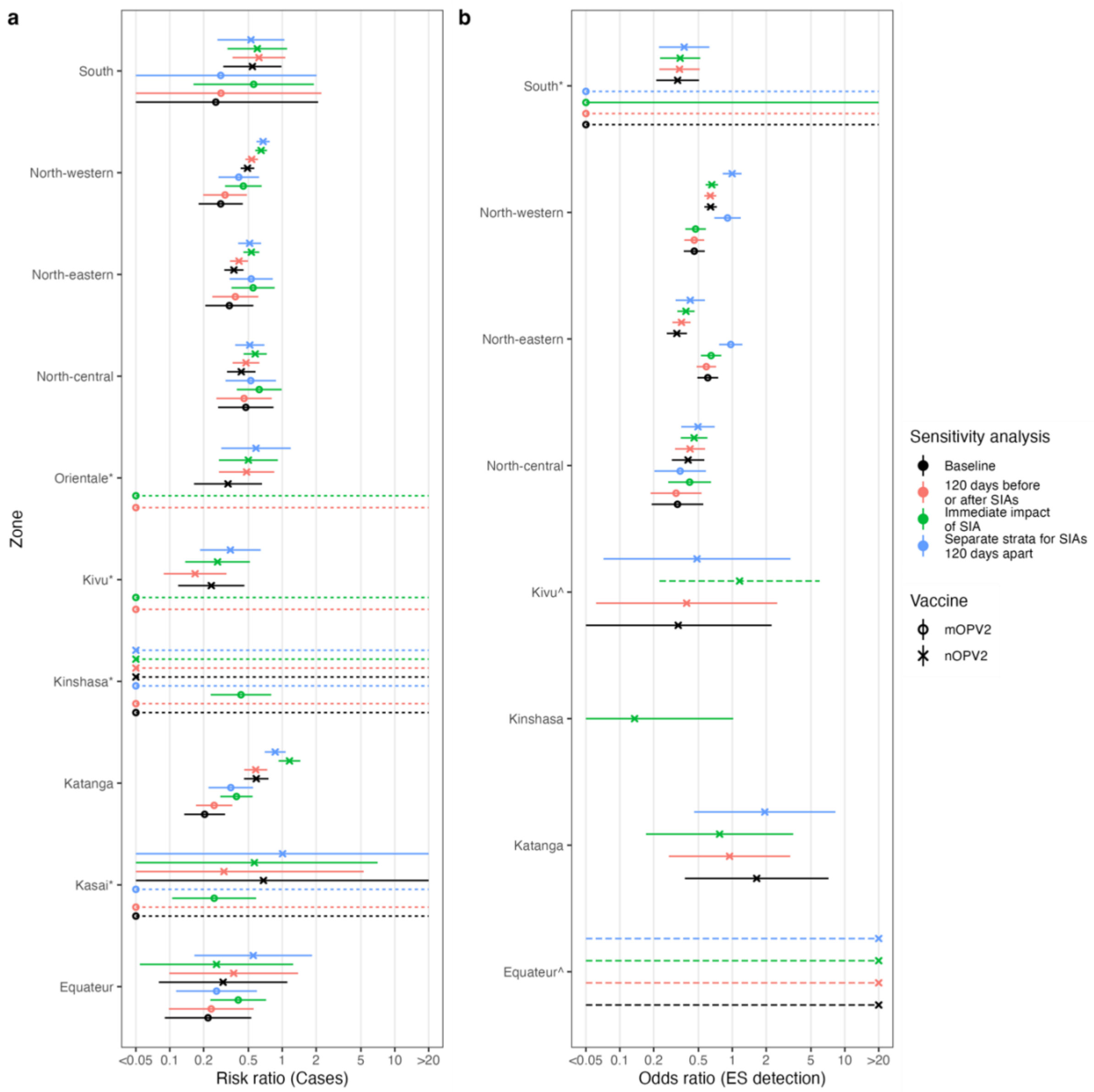

Figure S4. Sensitivity analyses for log-linear per-SIA impact of mOPV2 (circle) and nOPV2 (cross) on cVDPV2 incidence (risk ratio, RR) and prevalence in environmental surveillance (odds ratio, OR) in DRC and Nigeria by sub-national region, adjusting for immunity before the SIA. Points show central RR or OR estimate and vertical lines show 95% confidence interval (CI). Regions with an asterisk (*) and dotted error bars have zero cases or detections following SIAs. Regions with caret (^) and dashed error bars have zero cases or detections before an SIA.

Figure S5. Cumulative impact of one, two, three or four or more mOPV2 or nOPV2 SIAs on cVDPV2 incidence (risk ratio, RR), adjusting for immunity before the SIA. Points show central RR estimate and vertical lines show 95% confidence interval (CI). Where no point is shown, there is no stratum with this number of cumulative SIAs. Points with dashed error bars have zero cases after the SIA. Role of immunity: RR 1.09 (95% CI 1.06, 1.12) per 10% absolute increase.

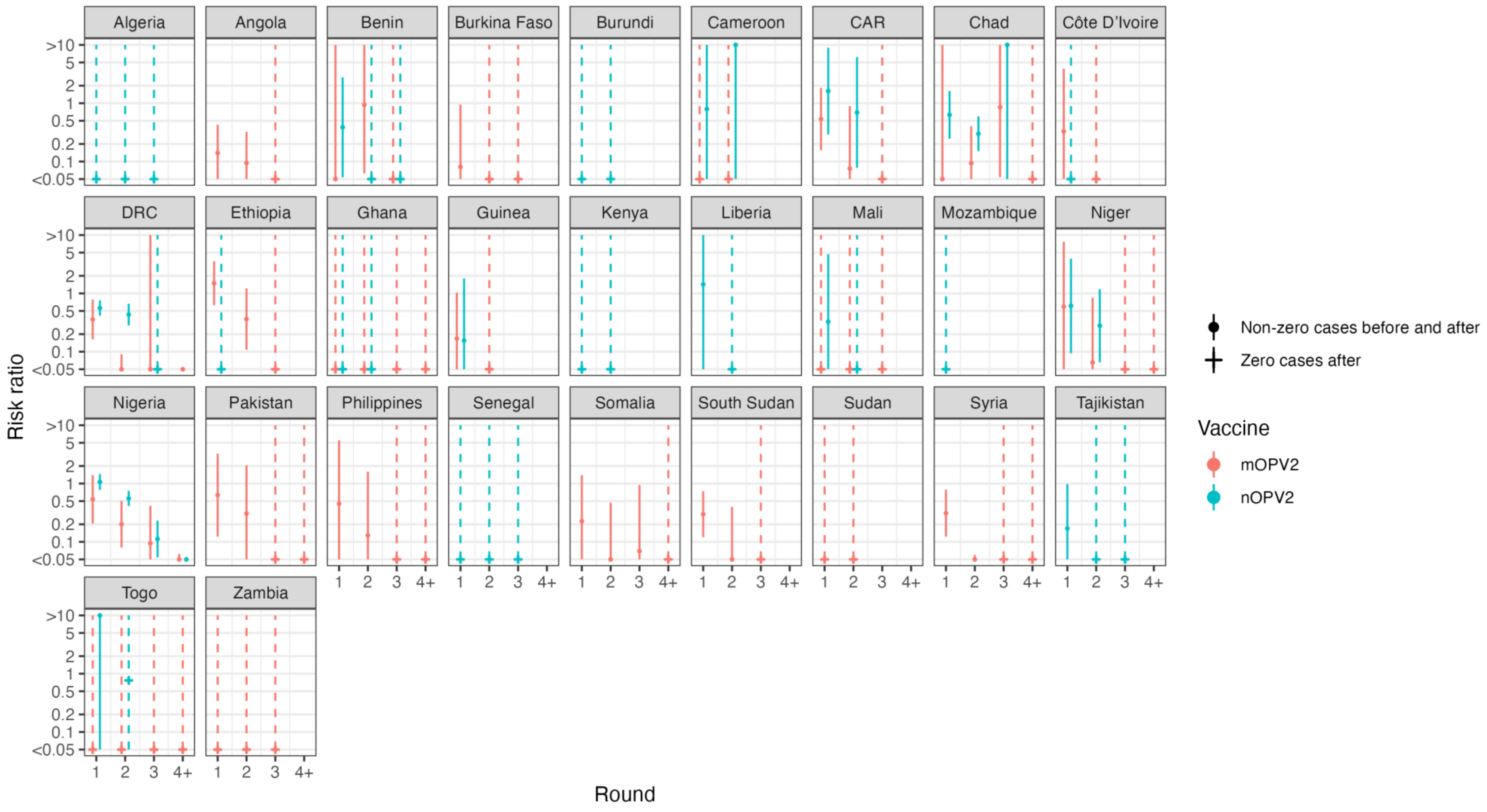

Figure S6. Cumulative impact of one, two, three or four or more mOPV2 or nOPV2 SIAs on cVDPV2 prevalence in environmental surveillance (odds ratio, OR), adjusting for immunity before the SIA. Points show central OR estimate and vertical lines show 95% confidence interval (CI). Where no point is shown, there is no stratum with this number of cumulative SIAs. Points with dashed error bars have zero detections before and/or after the SIA (distinguished by shape). Role of immunity: OR 1.06 (95% CI 1.04, 1.07) per 10% absolute increase.

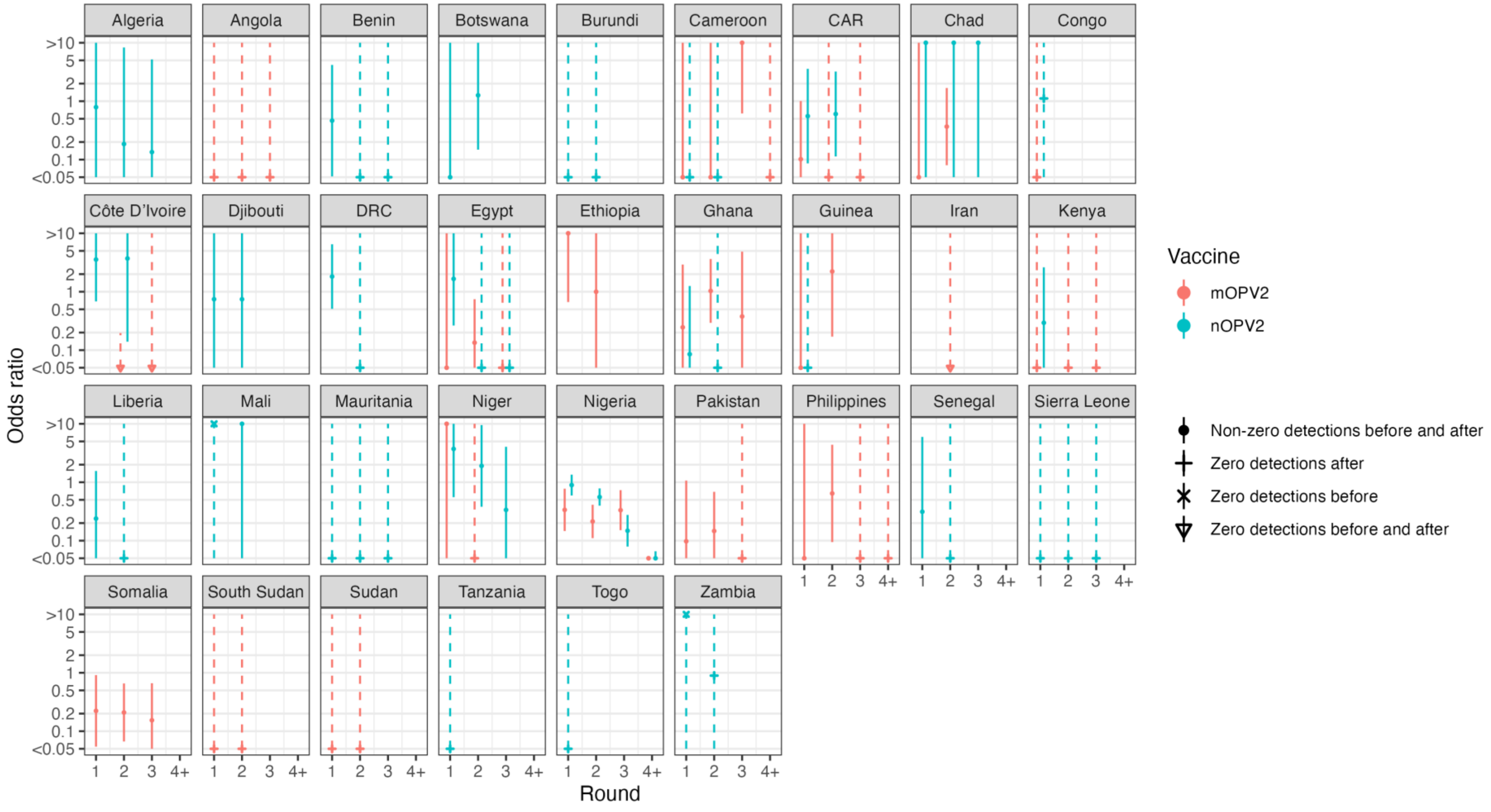

Figure S7. Cumulative impact of one, two, three or four or more mOPV2 or nOPV2 SIAs on cVDPV2 incidence (risk ratio, RR) or prevalence in environmental surveillance (odds ratio, OR), adjusting for immunity before the SIA. Points show central RR or OR estimate and vertical lines show 95% confidence interval (CI). Where no point is shown, there is no stratum with this number of cumulative SIAs. Points with dashed error bars have zero detections before and/or after the SIA (distinguished by shape). Lines and shaded ribbons indicate central estimate and 95% CI of log-linear model.

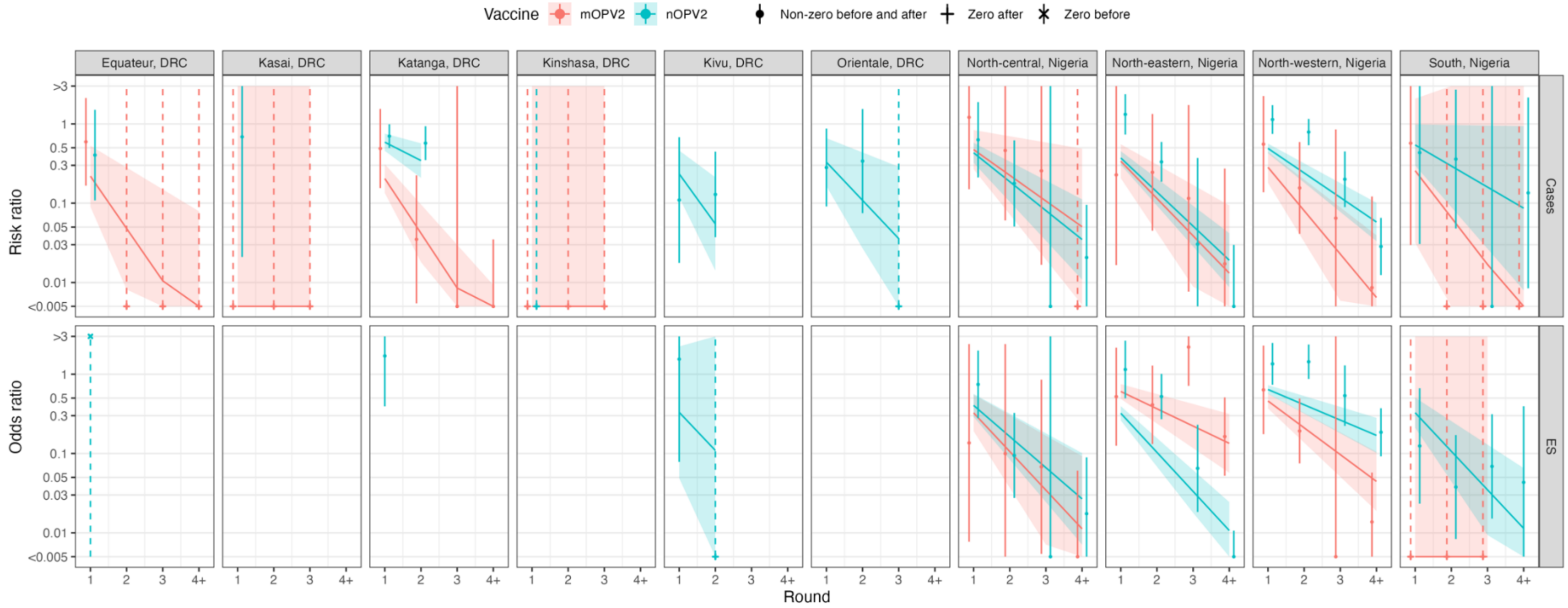

Supplementary results

Table S3. Number of strata included in incidence and prevalence analyses by country and vaccine type.

Country	Number of strata included in incidence analysis (cases)			Number of strata included in prevalence analysis (environmental surveillance)		
	nOPV2	mOPV2	Total	nOPV2	mOPV2	Total
Algeria	1	0	1	2	0	2
Angola	0	45	45	0	7	7
Benin	8	4	12	3	0	3
Botswana	0	0	0	3	0	3
Burkina Faso	0	14	14	0	0	0
Burundi	1	0	1	5	0	5
Cameroon	1	2	3	2	5	7
CAR	7	11	18	4	4	8
Chad	37	19	56	5	4	9
Congo	0	0	0	3[2]	3[2]	3
Côte D'Ivoire	3	13	16	10	7	17
Djibouti	0	0	0	1	0	1
DRC	96	53	149	9	0	9
Egypt	0	0	0	6	7	13
Ethiopia	2	14	16	0	3	3
Ghana	2	14	16	4	8	12
Guinea	3	11	14	1	2	3
Iran	0	0	0	0	2	2
Kenya	2	0	2	3	1	4
Liberia	2	0	2	3	0	3
Mali	4	8	12	1	0	1
Mauritania	0	0	0	1	0	1
Mozambique	2	0	2	0	0	0
Niger	10	3	13	7	1	8
Nigeria	223[1]	36[1]	254	103[3]	34[3]	131
Pakistan	0	9	9	0	13	13
Philippines	0	4	4	0	7	7
Senegal	1	0	1	2	0	2
Sierra Leone	0	0	0	3	0	3
Somalia	0	16	16	0	9	9
South Sudan	0	23	23	0	4	4
Sudan	0	12	12	0	2	2
Syria	0	5	5	0	0	0
Tajikistan	15	0	15	0	0	0
Tanzania	0	0	0	1	0	1
Togo	1	5	6	1	0	1
Zambia	0	1	1	1	0	1
All countries	416	317	738	175	114	298

[1]Five strata with both nOPV2 and mOPV2 SIAs in Nigeria. [2]Three strata with both nOPV2 and mOPV2 SIAs in Congo. [3]Six strata with both nOPV2 and mOPV2 SIAs in Nigeria.

Table S4. Statistical tests for model fits with and without adjustment for population immunity.

**Model**	**Geographic level**	**Difference between model with and without adjustment for immunity**
Incidence	Country-level	F-test p-value < 0.001
	Sub-national level	F-test p-value < 0.001
Prevalence	Country-level	AIC 2693 versus 2869
	Sub-national level	AIC 1658 versus 1724

Table S5. Log-linear per-SIA impact of mOPV2 and nOPV2 on cVDPV2 incidence (risk ratio, RR) across four regions of Nigeria, adjusting for immunity before the SIA and LQAS result. **Constrained variable.

**Factor**	**North-west**		**North-central**		**North-east**		**South**	
	aRR	95% CI	aRR	95% CI	aRR	95% CI	aRR	95% CI
mOPV2 SIA	0.243	(0.154-0.385)	0.583	(0.201-1.69)	0.468	(0.11-1.99)	0.374	(0.0367-3.82)
nOPV2 SIA	0.427	(0.352-0.517)	0.579	(0.264-1.27)	0.445	(0.201-0.986)	0.524	(0.209-1.31)
Fail	1.53	(1.03-2.26)	0.902	(0.251-3.24)	0.927	(0.331-2.59)	<0.001	(0-Inf)
No data	1.78	(1.31-2.42)	0.709	(0.182-2.77)	1.56	(0.969-2.51)	0.660	(0.106-4.11)
Pass	**	**	**	**	**	**	**	**
Immunity	1.15	(1.09-1.20)	1.06	(0.841-1.35)	1.05	(0.75-1.47)	1.42	(0.621-3.25)
**nOPV2 vs. mOPV2**								
Ratio	1.76		0.993		0.951		1.40	
P-value	0.0266		0.992		0.952		0.791	

Table S6. Log-linear per-SIA impact of mOPV2 and nOPV2 on cVDPV2 prevalence in ES (odds ratio, OR) across four regions of Nigeria, adjusting for immunity before the SIA and LQAS result. **Constrained variable.

**Factor**	**North-west**		**North-central**		**North-east**		**South**	
	aOR	95% CI	aRR	95% CI	aRR	95% CI	aRR	95% CI
mOPV2 SIA	0.320	(0.244-0.419)	0.2090	(0.0761-0.573)	0.360	(0.0491-2.64)	<0.001	(0-Inf)
nOPV2 SIA	0.477	(0.385-0.591)	0.0885	(0.0255-0.308)	0.256	(0.0726-0.9)	0.184	(0.0589-0.574)
Fail	1.40	(0.981-1.99)	0.630	(0.168-2.37)	0.370	(0.103-1.33)	1.10	(0.103-11.7)
No data	2.66	(1.75-4.06)	1.99	(0.486-8.14)	1.530	(0.567-4.13)	1.72	(0.425-6.99)
Pass	**	**	**	**	**	**	**	**
Immunity	1.10	(1.07-1.12)	2.08	(1.33-3.25)	1.200	(0.754-1.9)	1.21	(1.02-1.44)
**nOPV2 vs. mOPV2**								
Ratio	1.49		0.423		0.711		<0.001	
P-value	0.0231		0.294		0.777		1.00	

Table S7. Log-linear per-SIA impact of mOPV2 and nOPV2 on cVDPV2 incidence, adjusting for immunity before the first SIA. Role of immunity: RR 1.17 (95% CI 1.12, 1.21) per 10% absolute increase. Immunity is mean district-level type 2 vaccine-induced immunity in children 6-36 months of age 30 days prior to first SIA.

Vaccine	Country	Risk ratio	95% CI	Strata	Before SIA(s)		After SIA(s)		Immunity
					Cases	Person-years	Cases	Person-years	
mOPV2	Angola	0.213	0.123-0.37	45	92	382000	9	602000	1%
mOPV2	Benin	0.445	0.132-1.49	4	4	34200	1	54800	1%
mOPV2	Burkina Faso	0.0683	0.0072-0.648	14	19	198000	1	320000	0%
mOPV2	Cameroon	<0.001	<0.001->1000	2	2	3710	0	4550	1%
mOPV2	CAR	0.318	0.147-0.689	11	16	45000	6	83900	4%
mOPV2	Chad	0.316	0.167-0.599	19	27	49500	4	69700	3%
mOPV2	Côte D’Ivoire	0.144	0.0208-0.993	13	13	35000	1	43000	0%
mOPV2	DRC	0.193	0.137-0.272	53	98	270000	17	707000	19%
mOPV2	Ethiopia	0.477	0.312-0.729	14	19	558000	18	836000	7%
mOPV2	Ghana	<0.001	<0.001->1000	14	17	18000	0	25500	0%
mOPV2	Guinea	0.128	0.026-0.634	11	13	122000	2	238000	0%
mOPV2	Mali	<0.001	<0.001->1000	8	13	172000	0	202000	0%
mOPV2	Niger	0.202	0.0731-0.558	3	7	25500	2	70500	23%
mOPV2	Nigeria	0.33	0.247-0.442	36	32	380000	28	1610000	35%
mOPV2	Pakistan	0.476	0.226-1	9	11	399000	5	613000	1%
mOPV2	Philippines	0.343	0.119-0.987	4	10	95700	2	157000	0%
mOPV2	Somalia	0.223	0.106-0.471	16	15	104000	4	247000	29%
mOPV2	South Sudan	0.24	0.121-0.477	23	31	110000	10	221000	1%
mOPV2	Sudan	<0.001	<0.001->1000	12	14	42900	0	71200	0%
mOPV2	Syria	0.0821	0.044-0.153	5	46	15800	9	45400	56%
mOPV2	Togo	<0.001	<0.001->1000	5	7	22000	0	42000	0%
mOPV2	Zambia	<0.001	<0.001->1000	1	1	646	0	1060	0%
nOPV2	Algeria	<0.001	<0.001->1000	1	1	14800	0	30100	0%
nOPV2	Benin	0.265	0.0511-1.37	8	7	75200	2	79900	6%
nOPV2	Burundi	<0.001	<0.001->1000	1	1	10600	0	14200	0%
nOPV2	Cameroon	>1000	<0.001->1000	1	0	4610	1	7070	32%

Table S7 (ctd.). Log-linear per-SIA impact of mOPV2 and nOPV2 on cVDPV2 incidence, adjusting for immunity before the first SIA. Role of immunity: RR 1.17 (95% CI 1.12, 1.21) per 10% absolute increase. Immunity is mean district-level type 2 vaccine-induced immunity in children 6-36 months of age 30 days prior to first SIA.

Vaccine	Country	Risk ratio	95% CI	Strata	Before SIA(s)		After SIA(s)		Immunity
					Cases	Person-years	Cases	Person-years	
nOPV2	CAR	0.721	0.285-1.82	7	3	36700	8	56900	31%
nOPV2	Chad	0.426	0.299-0.607	37	34	116000	33	168000	40%
nOPV2	Côte D'Ivoire	<0.001	<0.001->1000	3	3	19300	0	3650	1%
nOPV2	DRC	0.505	0.409-0.623	96	177	717000	172	1010000	23%
nOPV2	Ethiopia	<0.001	<0.001->1000	2	2	65700	0	65500	17%
nOPV2	Ghana	<0.001	<0.001->1000	2	2	5450	0	7520	1%
nOPV2	Guinea	0.151	0.0134-1.69	3	18	87300	1	30100	4%
nOPV2	Kenya	<0.001	<0.001->1000	2	5	9170	0	4690	20%
nOPV2	Liberia	0.472	0.0487-4.58	2	1	3970	1	6700	0%
nOPV2	Mali	0.209	0.0219-2	4	4	26700	1	34200	11%
nOPV2	Mozambique	<0.001	<0.001->1000	2	3	9680	0	9570	0%
nOPV2	Niger	0.443	0.219-0.897	10	9	113000	6	144000	27%
nOPV2	Nigeria	0.47	0.42-0.526	223	165	3460000	353	16100000	33%
nOPV2	Senegal	<0.001	<0.001->1000	1	1	4640	0	16900	0%
nOPV2	Tajikistan	0.0765	0.019-0.308	15	27	87900	2	161000	35%
nOPV2	Togo	0.88	0.0584-13.3	1	0	6440	1	10600	8%

Table S8. Log-linear per-SIA impact of mOPV2 and nOPV2 on cVDPV2 prevalence in ES, adjusting for immunity before the first SIA. Role of immunity: OR 1.12 (95% CI 1.10, 1.14) per 10% absolute increase. Immunity is mean district-level type 2 immunity in children 6-36 months of age 30 days prior to first SIA.

Vaccine	Country	Odds ratio	95% CI	Strata	Samples before SIA(s)		Samples after SIA(s)		Immunity
					Positive	Total	Positive	Total	
mOPV2	Angola	<0.001	<0.001->1000	7	15	23	23	27	0%
mOPV2	Cameroon	0.731	0.434-1.23	5	4	24	24	34	5%
mOPV2	CAR	0.0704	0.00964-0.514	4	9	17	17	29	10%
mOPV2	Chad	0.551	0.244-1.25	4	7	24	24	30	3%
mOPV2	Congo	<0.001	<0.001->1000	3	3	24	24	40	0%
mOPV2	Côte D'Ivoire	0.109	0.0306-0.391	7	19	21	21	25	0%
mOPV2	Egypt	0.268	0.112-0.638	7	9	30	30	64	0%
mOPV2	Ethiopia	0.999	0.303-3.3	3	1	9	9	12	0%
mOPV2	Ghana	0.864	0.517-1.44	8	11	37	37	43	5%
mOPV2	Guinea	1.84	0.369-9.18	2	1	12	12	26	0%
mOPV2	Iran	<0.001	<0.001->1000	2	2	6	6	6	0%
mOPV2	Kenya	<0.001	<0.001->1000	1	1	12	12	13	34%
mOPV2	Niger	0.199	0.0233-1.71	1	2	3	3	5	0%
mOPV2	Nigeria	0.405	0.345-0.475	34	41	174	174	804	43%
mOPV2	Pakistan	0.259	0.121-0.553	13	17	50	50	55	16%
mOPV2	Philippines	0.57	0.267-1.22	7	6	17	17	32	0%
mOPV2	Somalia	0.385	0.246-0.604	9	14	63	63	135	49%
mOPV2	South Sudan	<0.001	<0.001->1000	4	4	8	8	24	0%
mOPV2	Sudan	<0.001	<0.001->1000	2	3	6	6	8	0%
nOPV2	Algeria	0.478	0.167-1.37	2	3	4	4	26	0%
nOPV2	Benin	0.256	0.049-1.34	3	4	11	11	22	15%
nOPV2	Botswana	1.12	0.361-3.49	3	2	17	17	19	0%
nOPV2	Burundi	<0.001	<0.001->1000	5	7	15	15	18	0%
nOPV2	Cameroon	<0.001	<0.001->1000	2	5	11	11	10	47%
nOPV2	CAR	0.722	0.306-1.7	4	4	22	22	39	17%
nOPV2	Chad	0.78	0.281-2.17	5	0	10	10	52	39%
nOPV2	Congo	1.11	<0.001->1000	3	3	46	46	18	0%
nOPV2	Côte D'Ivoire	2.08	0.525-8.24	10	13	44	44	22	42%

Table S8 (ctd.). Log-linear per-SIA impact of mOPV2 and nOPV2 on cVDPV2 prevalence in ES, adjusting for immunity before the first SIA. Role of immunity: OR 1.12 (95% CI 1.10, 1.14) per 10% absolute increase. Immunity is mean district-level type 2 immunity in children 6-36 months of age 30 days prior to first SIA.

Vaccine	Country	Odds ratio	95% CI	Strata	Samples before SIA(s)		Samples after SIA(s)		Immunity
					Positive	Total	Positive	Total	
nOPV2	Djibouti	0.591	<0.001->1000	1	5	5	5	2	1%
nOPV2	DRC	0.857	0.292-2.52	9	6	51	51	53	21%
nOPV2	Egypt	0.375	0.125-1.13	6	3	29	29	41	51%
nOPV2	Ghana	0.0626	0.00593-0.662	4	6	11	11	20	9%
nOPV2	Guinea	<0.001	<0.001->1000	1	1	4	4	2	4%
nOPV2	Kenya	0.26	0.0293-2.32	3	6	12	12	7	20%
nOPV2	Liberia	0.132	0.0309-0.565	3	10	22	22	28	0%
nOPV2	Mali	3.83	0.321-45.7	1	0	3	3	4	12%
nOPV2	Mauritania	<0.001	<0.001->1000	1	2	11	11	35	0%
nOPV2	Niger	0.712	0.4-1.27	7	3	40	40	90	39%
nOPV2	Nigeria	0.475	0.424-0.531	103	104	388	388	2072	35%
nOPV2	Senegal	0.202	0.0186-2.19	2	1	6	6	30	0%
nOPV2	Sierra Leone	<0.001	<0.001->1000	3	3	38	38	71	0%
nOPV2	Tanzania	<0.001	<0.001->1000	1	1	3	3	1	0%
nOPV2	Togo	<0.001	<0.001->1000	1	1	6	6	6	24%
nOPV2	Zambia	0.753	0.0663-8.56	1	0	3	3	8	0%

Table S9. Log-linear per-SIA impact of mOPV2 and nOPV2 on cVDPV2 incidence in sub-national regions of DRC and Nigeria, adjusting for immunity before the first SIA. Role of immunity: RR 1.17 (95% CI 1.12, 1.22) per 10% absolute increase. Immunity is mean district-level type 2 immunity in children 6-36 months of age 30 days prior to first SIA.

Vaccine	Country	Region	Risk ratio	95% CI	Strata	Before SIA(s)		After SIA(s)		Immunity
						Cases	Person-years	Cases	Person-years	
mOPV2	DRC	Equateur	0.219	0.0904-0.53	13	18	43100	5	71900	12%
mOPV2	DRC	Kasai	<0.001	<0.001->1000	12	32	44700	0	54700	1%
mOPV2	DRC	Katanga	0.204	0.134-0.31	14	26	141000	12	531000	60%
mOPV2	DRC	Kinshasa	<0.001	<0.001->1000	14	22	41000	0	49500	1%
mOPV2	Nigeria	North-central	0.474	0.269-0.835	7	6	90800	5	249000	11%
mOPV2	Nigeria	North-eastern	0.338	0.207-0.553	10	8	78700	6	234000	40%
mOPV2	Nigeria	North-western	0.284	0.181-0.446	15	15	192000	16	1070000	52%
mOPV2	Nigeria	South	0.257	0.0316-2.08	4	3	19300	1	51800	1%
nOPV2	DRC	Equateur	0.298	0.08-1.11	7	9	19600	6	24000	39%
nOPV2	DRC	Kasai	0.68	0.0214-21.7	2	1	15800	1	22900	1%
nOPV2	DRC	Katanga	0.587	0.457-0.754	57	128	576000	147	784000	18%
nOPV2	DRC	Kinshasa	<0.001	<0.001->1000	1	1	4460	0	545	2%
nOPV2	DRC	Kivu	0.234	0.119-0.46	15	22	56600	7	91200	36%
nOPV2	DRC	Orientale	0.329	0.164-0.66	14	16	44700	11	90300	23%
nOPV2	Nigeria	North-central	0.432	0.323-0.578	19	20	335000	16	1470000	27%
nOPV2	Nigeria	North-eastern	0.372	0.305-0.452	50	46	705000	67	3700000	23%
nOPV2	Nigeria	North-western	0.491	0.427-0.564	143	92	2310000	266	10700000	39%
nOPV2	Nigeria	South	0.542	0.299-0.984	11	7	107000	4	273000	11%

Table S10. Log-linear per-SIA impact of mOPV2 and nOPV2 on cVDPV2 prevalence in ES in sub-national regions of DRC and Nigeria, adjusting for immunity before the first SIA. Role of immunity: OR 1.08 (95% CI 1.06, 1.11) per 10% absolute increase. Immunity is mean district-level type 2 immunity in children 6-36 months of age 30 days prior to first SIA.

Vaccine	Country	Region	Odds ratio	95% CI	Strata	Samples before SIA(s)		Samples after SIA(s)		Immunity
						Positive	Total	Positive	Total	
mOPV2	Nigeria	North-central	0.326	0.192-0.552	3	8	11	11	52	5%
mOPV2	Nigeria	North-eastern	0.605	0.489-0.749	11	11	69	69	235	47%
mOPV2	Nigeria	North-western	0.459	0.371-0.569	16	15	82	82	537	61%
mOPV2	Nigeria	South	<0.001	<0.001->1000	5	8	22	22	30	10%
nOPV2	DRC	Equateur	>1000	<0.001->1000	1	0	5	5	8	42%
nOPV2	DRC	Katanga	1.65	0.379-7.16	6	5	36	36	28	12%
nOPV2	DRC	Kivu	0.331	0.0489-2.23	2	1	10	10	17	36%
nOPV2	Nigeria	North-central	0.405	0.29-0.564	16	17	65	65	322	23%
nOPV2	Nigeria	North-eastern	0.322	0.262-0.395	23	28	75	75	568	26%
nOPV2	Nigeria	North-western	0.641	0.565-0.729	48	41	193	193	1003	49%
nOPV2	Nigeria	South	0.327	0.211-0.506	16	18	55	55	179	22%

Table S11. Log-linear per-SIA impact of mOPV2 and nOPV2 on cVDPV2 incidence, adjusting for vaccine-induced population immunity before the first SIA separately for mOPV2 and nOPV2 SIAs.

Factor	Vaccine	Country	Risk ratio	95% CI
10% absolute increase in immunity	mOPV2	-	1.18	1.08-1.29
10% absolute increase in immunity	nOPV2	-	1.16	1.12-1.21
SIA	mOPV2	Angola	0.213	0.123-0.368
SIA	mOPV2	Benin	0.444	0.133-1.48
SIA	mOPV2	Burkina Faso	0.0683	0.00726-0.642
SIA	mOPV2	Cameroon	<0.001	<0.001->1000
SIA	mOPV2	CAR	0.317	0.146-0.684
SIA	mOPV2	Chad	0.314	0.166-0.594
SIA	mOPV2	Côte D'Ivoire	0.144	0.0209-0.985
SIA	mOPV2	DRC	0.182	0.108-0.308
SIA	mOPV2	Ethiopia	0.471	0.305-0.727
SIA	mOPV2	Ghana	<0.001	<0.001->1000
SIA	mOPV2	Guinea	0.128	0.0261-0.63
SIA	mOPV2	Mali	<0.001	<0.001->1000
SIA	mOPV2	Niger	0.195	0.0685-0.552
SIA	mOPV2	Nigeria	0.317	0.212-0.475
SIA	mOPV2	Pakistan	0.476	0.227-0.999
SIA	mOPV2	Philippines	0.343	0.12-0.982
SIA	mOPV2	Somalia	0.216	0.0985-0.472
SIA	mOPV2	South Sudan	0.24	0.121-0.476
SIA	mOPV2	Sudan	<0.001	<0.001->1000
SIA	mOPV2	Syria	0.0769	0.0359-0.165
SIA	mOPV2	Togo	<0.001	<0.001->1000
SIA	mOPV2	Zambia	<0.001	<0.001->1000
SIA	nOPV2	Benin	0.265	0.0515-1.37
SIA	nOPV2	Burundi	<0.001	<0.001->1000
SIA	nOPV2	Cameroon	>1000	<0.001->1000
SIA	nOPV2	CAR	0.727	0.288-1.83
SIA	nOPV2	Chad	0.431	0.301-0.618
SIA	nOPV2	Côte D'Ivoire	<0.001	<0.001->1000
SIA	nOPV2	DRC	0.509	0.41-0.632
SIA	nOPV2	Ethiopia	<0.001	<0.001->1000
SIA	nOPV2	Ghana	<0.001	<0.001->1000
SIA	nOPV2	Guinea	0.151	0.0136-1.68
SIA	nOPV2	Kenya	<0.001	<0.001->1000
SIA	nOPV2	Liberia	0.472	0.0491-4.53
SIA	nOPV2	Mali	0.21	0.0222-1.98
SIA	nOPV2	Mozambique	<0.001	<0.001->1000
SIA	nOPV2	Niger	0.446	0.221-0.903
SIA	nOPV2	Nigeria	0.473	0.419-0.535
SIA	nOPV2	Senegal	<0.001	<0.001->1000
SIA	nOPV2	Tajikistan	0.0772	0.0192-0.31
SIA	nOPV2	Togo	0.882	0.0592-13.1

Table S12. Log-linear per-SIA impact of mOPV2 and nOPV2 on cVDPV2 prevalence in environmental surveillance, adjusting for vaccine-induced population immunity before the first SIA separately for mOPV2 and nOPV2 SIAs.

Factor	Vaccine	Country	Odds ratio	95% CI
10% absolute increase in immunity	mOPV2	-	1.1	1.04-1.16
10% absolute increase in immunity	nOPV2	-	1.12	1.1-1.15
SIA	mOPV2	Angola	<0.001	<0.001->1000
SIA	mOPV2	Cameroon	0.742	0.439-1.25
SIA	mOPV2	CAR	0.0712	0.00971-0.522
SIA	mOPV2	Chad	0.554	0.245-1.25
SIA	mOPV2	Congo	<0.001	<0.001->1000
SIA	mOPV2	Côte D'Ivoire	0.109	0.0306-0.391
SIA	mOPV2	Egypt	0.268	0.112-0.639
SIA	mOPV2	Ethiopia	0.999	0.303-3.3
SIA	mOPV2	Ghana	0.866	0.518-1.45
SIA	mOPV2	Guinea	1.84	0.369-9.18
SIA	mOPV2	Iran	<0.001	<0.001->1000
SIA	mOPV2	Kenya	<0.001	<0.001->1000
SIA	mOPV2	Niger	0.199	0.0233-1.71
SIA	mOPV2	Nigeria	0.44	0.32-0.604
SIA	mOPV2	Pakistan	0.267	0.125-0.573
SIA	mOPV2	Philippines	0.57	0.267-1.22
SIA	mOPV2	Somalia	0.412	0.249-0.682
SIA	mOPV2	South Sudan	<0.001	<0.001->1000
SIA	mOPV2	Sudan	<0.001	<0.001->1000
SIA	nOPV2	Benin	0.255	0.0488-1.33
SIA	nOPV2	Botswana	1.12	0.361-3.49
SIA	nOPV2	Burundi	<0.001	<0.001->1000
SIA	nOPV2	Cameroon	<0.001	<0.001->1000
SIA	nOPV2	CAR	0.72	0.305-1.7
SIA	nOPV2	Chad	0.771	0.277-2.15
SIA	nOPV2	Congo	1.11	<0.001->1000
SIA	nOPV2	Côte D'Ivoire	2.06	0.519-8.15
SIA	nOPV2	Djibouti	0.591	<0.001->1000
SIA	nOPV2	DRC	0.852	0.29-2.5
SIA	nOPV2	Egypt	0.37	0.123-1.11
SIA	nOPV2	Ghana	0.0625	0.00591-0.661
SIA	nOPV2	Guinea	<0.001	<0.001->1000
SIA	nOPV2	Kenya	0.259	0.0291-2.3
SIA	nOPV2	Liberia	0.132	0.0309-0.565
SIA	nOPV2	Mali	3.82	0.32-45.6
SIA	nOPV2	Mauritania	<0.001	<0.001->1000
SIA	nOPV2	Niger	0.706	0.396-1.26
SIA	nOPV2	Nigeria	0.469	0.417-0.529
SIA	nOPV2	Senegal	0.202	0.0186-2.19
SIA	nOPV2	Sierra Leone	<0.001	<0.001->1000
SIA	nOPV2	Tanzania	<0.001	<0.001->1000
SIA	nOPV2	Togo	<0.001	<0.001->1000
SIA	nOPV2	Zambia	0.753	0.0663-8.56

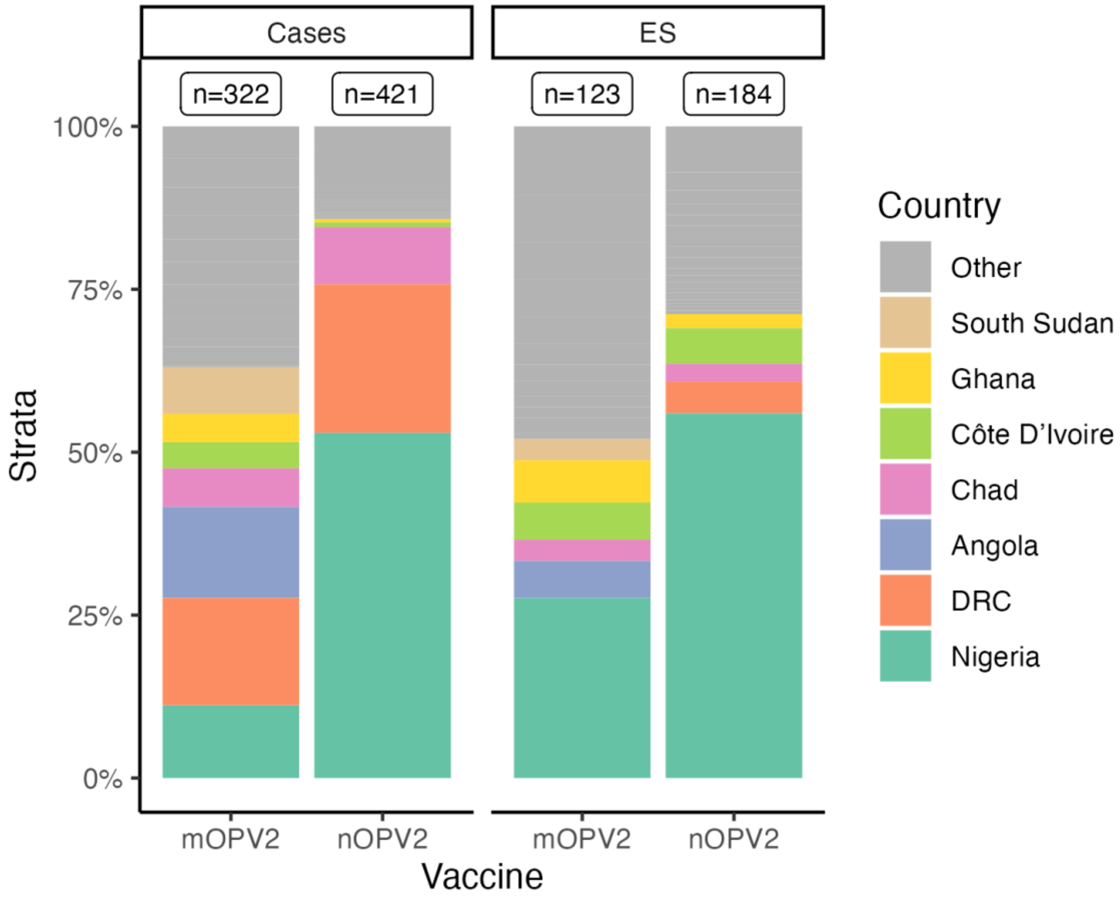

Figure S8. Proportion of strata by analysis, vaccine, and country. Seven most frequent countries identified and less frequent countries aggregated into "Other". Total strata with mOPV2 or nOPV2 in incidence

References

1. Voorman A, Lyons H, Bennette C, Kovacs S, Makam JK, F Vertefeuille J, Tallis G. Analysis of population immunity to poliovirus following cessation of trivalent oral polio vaccine. Vaccine. 2023 Apr 6;41 Suppl 1(Suppl 1):A85-A92. doi: 10.1016/j.vaccine.2022.03.013. Epub 2022 Mar 23. PMID: 35339308; PMCID: PMC10973941
2. United Nations, Department of Economic and Social Affairs, Population Division (2022). World Population Prospects 2022: Methodology of the United Nations population estimates and projections (UN DESA/POP/2022/TR/NO. 4).
3. Tatem AJ, Garcia AJ, Snow RW, Noor AM, Gaughan AE, Gilbert M, Linard C. Millennium development health metrics: where do Africa's children and women of childbearing age live?. Population health metrics. 2013 Dec;11:1-1.
4. Armstrong, B.G., Gasparrini, A. & Tobias, A. Conditional Poisson models: a flexible alternative to conditional logistic case cross-over analysis. *BMC Med Res Methodol* **14**, 122 (2014). https://doi.org/10.1186/1471-2288-14-122
5. Heather Turner and David Firth (2022). Generalized nonlinear models in R: An overview of the gnm package. (R package version 1.1-2). (https://cran.r-project.org/package=gnm).
6. Venables WN, Ripley BD. Modern applied statistics with S-PLUS. Springer Science & Business Media; 2013 Apr 17.
